# Using spatial mark-recapture for conservation monitoring of grizzly bear populations in Alberta

**DOI:** 10.1038/s41598-018-23502-3

**Published:** 2018-03-26

**Authors:** John Boulanger, Scott E. Nielsen, Gordon B. Stenhouse

**Affiliations:** 1 Integrated Ecological Research, 924 Innes St., Nelson, BC V1L 5T2 Canada; 2 Department of Renewable Resources, University of Alberta, 751 General Services Building, Edmonton, AB T6G 2H1 Canada; 3 fRI Research 1176 Switzer Drive, Hinton, Alberta T7V 1V3 Canada

## Abstract

One of the challenges in conservation is determining patterns and responses in population density and distribution as it relates to habitat and changes in anthropogenic activities. We applied spatially explicit capture recapture (SECR) methods, combined with density surface modelling from five grizzly bear (*Ursus arctos*) management areas (BMAs) in Alberta, Canada, to assess SECR methods and to explore factors influencing bear distribution. Here we used models of grizzly bear habitat and mortality risk to test local density associations using density surface modelling. Results demonstrated BMA-specific factors influenced density, as well as the effects of habitat and topography on detections and movements of bears. Estimates from SECR were similar to those from closed population models and telemetry data, but with similar or higher levels of precision. Habitat was most associated with areas of higher bear density in the north, whereas mortality risk was most associated (negatively) with density of bears in the south. Comparisons of the distribution of mortality risk and habitat revealed differences by BMA that in turn influenced local abundance of bears. Combining SECR methods with density surface modelling increases the resolution of mark-recapture methods by directly inferring the effect of spatial factors on regulating local densities of animals.

## Introduction

One of the key challenges in conservation and management of threatened species is determining which factors most influence their abundance and thus being targets for monitoring and recovery actions. This is especially challenging for wide-ranging, generalist species such as grizzly bears (*Ursus arctos*), which have highly mobility and complex interactive relationships between anthropogenic disturbances, habitats, and mortality risk. First, widespread movements of bears can result in closure bias complicating interpretation of traditional closed methods of mark-recapture abundance estimates^1,2^. Second, anthropogenic disturbances can paradoxically increase local habitat value attracting bears to areas of higher mortality risk that result in population sinks^3–5^ and reduced population viability^6,7^. Temporal and spatial scales interact so that the current distribution of bears is a result of both historic and current landscape conditions, as well as human perceptions and tolerance towards bears^8,9^. All of these factors complicate the interpretation of grizzly bear population status based on single estimates of abundance.

Large-scale DNA mark-recapture inventories were completed for 5 of 7 bear management areas (BMAs) in Alberta from 2004–8^2,10,11^ representing an overall area of 48,229 km^2^. BMAs ranged in size from 2,827 to 19,502 km^2^ (Table 1), obscuring local variation in density and importantly its potential causes. To address this variation, resource selection function models have been applied to frequencies of detection at DNA sites to model potential distribution of grizzly bears within individual study areas^12^. While this approach has produced useful predictions of occurrence and potential local density, the actual scale of habitat selection is not estimated from observed movements of bears during the DNA survey given that data from individual bear movements was not used in the analysis. Likewise, analyses based solely on frequencies of detection at hair snag sites ignore the influence of differences in detection probabilities among hair snag sites which may vary from that of habitat selection^13^.

**Table 1:** Dimensions of Alberta DNA sample grids as defined by cells (49 km^2^) used to systematically place DNA hair snag sites, % protected area in each DNA grid, bears detected in DNA sampling, proportion of bears detected in more than 1 sampling session (p > 1) and collared bears available for joint closed model/telemetry analyses for analysis of Alberta grizzly bear inventory data (2004–8).

BMA	Name	Year	Area (km^2^)	% protected	Bears detected			Efficiency	Collared bears	
					F	M	Total	P (>1)	Female	Male
2	Grande Cache	2008	19,502	50.0%	161	108	269	0.45	23	37
3	Yellowhead	2004	8,820	2.5%	24	20	44	0.57	38	33
4	Clearwater	2005	9,016	7.5%	25	17	42	0.71	11	2
5	Livingston	2006	8,134	44.3%	45	40	85	0.42	19	18
6	Castle	2007	2,827	17.7%	13	19	32	0.19	6	4

Appendix S1 provides further details on sampling for each BMA.

Recent advances in spatially explicit mark-recapture methods (SECR) have the potential to provide more robust estimates of populations without the use of collared animals to estimate scale of movement relative to sampling^14^, while also providing inference around factors affecting local variation in density using density surface modelling^15,16^. This approach models the distribution of estimated home range centers within the sample grid using scale of movement parameters estimated from repeat detections of animals during the time of sampling. The actual scale of movements and associated selection is therefore incorporated directly into the analysis. Covariates that describe factors influencing detection at sites can also be added to the model to reduce the potential effect of confounding from differences in detectability at DNA sites and local variation in habitat selection.

In this paper we use SECR methods of grizzly bear DNA sampling for most of the range of grizzly bears in Alberta^17–19^. We first compare SECR estimates with previous estimates using traditional techniques that use radio-collared bears and closed mark-recapture models. We then examine local variation in density using estimated home range centers of bears on the sampling grid, as well as density surface models that utilize previously developed resource selection function (RSF) and mortality risk models^5^. Our ultimate objective is to assess whether the distribution of bear home ranges is more associated with habitat (RSF) or mortality risk (Risk) among different grizzly bear BMAs in Alberta thereby providing inference on larger-scale factors that may limit grizzly bear populations within sampling grids. The general approach is analogous to the testing of “bottom up versus top down” models in ecology^20^. The density surface modelling used in this manuscript should be applicable to other species that exhibit large-scale variation in density across sampled areas.

## Methods

### Study area

**Figure 1:**
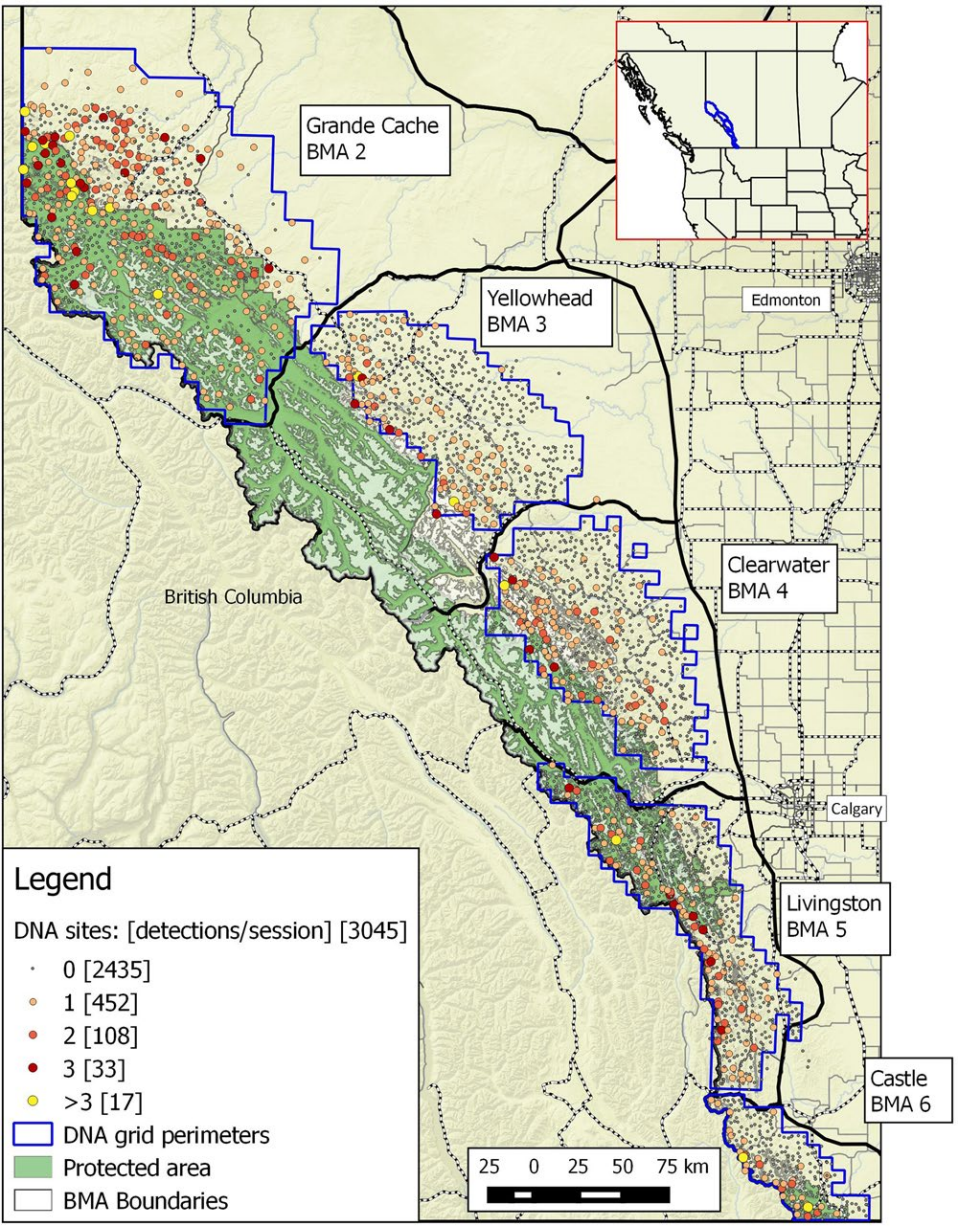
Distribution of DNA hair snag sites in Alberta, Canada by each bear management area (BMA) sampled from 2004–8. Sites are categorized by the mean number of bears detected per session. Frequency of each detection/session category is provided in brackets. Grid perimeters are shown delineating the extent of areas sampled. A minimum of one site was placed in each 49 km^2^ cell for each DNA grid. Map was produced using QGIS software (v2.10.1; qgis.org).

Our study area was divided into five grizzly BMAs in Alberta, Canada (Table 1 and Fig. 1) where the topography varies from plains and foothills to subalpine and high alpine areas. Previous research demonstrated that major highway corridors limited movements of females and partially defined boundaries of different BMAs^9^. A history of forest fires, forest harvesting, mining, energy exploration and development has created a mosaic of different forest types and stand ages, indicated by patterns in regenerating forest habitats and an array of permanent road networks^5,21–23^. A portion of each DNA sample grid was in high elevation rock and ice which is not considered viable habitat for grizzly bears. To address this, we estimated the proportion of barren landcover at elevations of greater than 2000 m in each grid cell. Our study area also included federal and provincial parks and protected areas, including parts of Jasper National Park, Banff National Park, and other protected areas where anthropogenic changes in habitat are uncommon and motorized road access limited to only a few areas (most often being valley bottoms).

The majority of grizzly bear habitat in each BMA was sampled using a systematic design in which one hair snag station was placed in a 7 × 7 km grid cell and sampled for 4 sessions. Hair snag sites were composed of a pile of small logs and sticks with a liquid lure surrounded by a single strand of barbed wire laid out as a corral^2,10^. Grizzly bear habitat selection likely occurs at scales close to the 7 × 7 km cell level^12^ and therefore the objective of site selection was to place sites in micro-habitats within cells that bears may traverse to maximize site encounter^2,24^. Site selection was done prior to fieldwork and based on grizzly bear RSF models that had been developed for each individual BMA^5,25^, GPS collar locations, remote sensing-based habitat mapping^26^, aerial photographs, and expert opinion of bear biologists^2^. In most cases, each hair capture site was moved after each session to ensure better coverage of each grid cell while sampling a range of habitats available in each cell. Previous research in the area demonstrated that a design with sites moved and with one strand of barbed wire was suitable for mark-recapture sampling^10^. Transect grid cells were used in BMAs 2, 3, 4, and 5 where a small number of sites were placed to the east of the main study grid to identify occupancy limits along the eastern periphery of the grizzly bear range. Percent of protected areas within each DNA grid varied from 2.5% in BMA 3 to 50% in BMA 2.

We also utilized data from radio collared bears in each BMA (Table 1)^27^ for closed model/telemetry analyses. All collared bears were live captured in accordance with Canadian Council on Animal Care and American Society of Mammalogists animal care guidelines^28–30^. The animal care guidelines and experimental protocols for our research received annual approval from the University of Saskatchewan and the Government of Alberta through research permit applications.

### Analysis methods

#### Spatially explicit analysis of detection and movement

Spatially explicit capture-recapture (SECR) methods^31–34^, also known as spatially explicit mark-recapture methods, were used to estimate grizzly bear population size and density. Spatially explicit methods estimate the spatial scale of movement relative to sites for bears that are detected repeatedly. Unlike closed models that pool data from multiple hair snag sites within each session for each bear, the SECR method uses multiple detections of bears at unique hair snag sites within a session to model bear movements and detection probabilities. Using this information, we estimated the detection probabilities of grizzly bears at their home range center (*g*_0_), spatial scale of grizzly bear movements (σ) around home range centers, and bear density. An assumption of this method is that the grizzly bear home range can be approximated by a circular symmetrical distribution of use (Efford 2004). The shape and configuration of the sampling grid was used in the process of estimating home ranges, scale of movements, and density, therefore accounting for the effect of study-area size and configuration on the degree of closure violation and subsequent density estimates.

We chose the sampling grid as defined by the DNA grid perimeters to be our main unit of inference for estimation of density (Fig. 1). This makes estimates equitable with closed model/telemetry methods. SECR methods use a mask which is a set of systematic points that cover the grid and surrounding areas that might contain home range centers of animal sampled on the grid. Density is then estimated for each mask point. To estimate the size of the mask relative to study area size needed to minimize bias in density estimates, the *esa.plot* function in program *secr*^35^ was run for sex-specific *g*_0_ and σ models for each BMA. Spacing of SECR mask centroids were 3.5 km on all DNA grids. Sensitivity analyses suggested that this spacing optimized computation time with minimal changes in estimates compared to tighter spacing of mask centroids.

We ran the analysis in two phases. In the first phase, we fit detection and scale of movement models for each sex of bear and BMA. These models included temporal variation, behavioural responses at the individual bear and at DNA sites, and undefined heterogeneity variation (as modelled by Pledger mixture models^36^). Because sites were moved between sessions for the majority of projects, the applicability of site-specific behavioural response models was limited. To describe variation in detection probabilities at the home range center and scale of movement, environmental covariates around hair snag sites (site and home range scales) were also measured using terrain ruggedness (*TRI*)^37^ and canopy closure (*CC*). Selection of these two variables, which describe the general topography and degree of openness around sites, were based on the field experience of researchers and other studies^13^. Scale in this case was based on the extent in which habitat variables were summarized in a GIS relative to hair snag sites. The landscape scale was based on an extent of 10 kilometers surrounding hair snag sites which corresponded to average bear home ranges^38^, while the site scale was based on a 1.69-km radius that represents the scale of attraction of hair snag sites from previous analysis of radio collared bear data^39^. Density was assumed to be constant across the extent of the survey area for this analysis. Information theoretic methods^40^ were then used to evaluate relative support of models. Further details regarding the SECR analysis, including figures that show SECR masks used for each BMA analysis, are provided in Appendix S1.

#### Closed model and telemetry analyses

We also wanted to determine whether density estimates from SECR models were similar to those derived from historic closed model and telemetry analyses, as well as the Closed N/Telemetry density estimator in program MARK^41^. This historic closed model with telemetry estimates were derived using proportion of points of collared bears on the sample grid to estimate overall residency of bears^42^ (BMAs 3 and 4) and is a precursor to the Closed N/Telemetry estimator^43,44^, which is based on distance of radio collared bears from the grid edge^1^. We used radio collar data from the same time period of sampling in years previous and after the DNA surveys to estimate residency as a function of mean location of collared bears from the grid edge for each BMA. Only locations from within the DNA grids were used to define mean location of collared bears for the density analysis to ensure equivalence with DNA-based mean detection locations. The closed N/Telemetry analysis used detection models based on previous closed models. In these analyses, the most supported Huggins detection models were used for each BMA^17–19^. Further details on the MARK analysis are given in Appendix S2.

#### Density surface modelling of distribution

Density on the sample grids potentially varies due to habitat selection^5^, road densities, and associated risk of mortality^6,45^. A central question of our analyses was determining how these factors influence and interact to change distribution of grizzly bears detected during DNA sampling.

As an initial assessment of distribution, home range centers were predicted using the most supported model on detection and scale of movement from phase 1 of the analysis^46,47^. This approach uses the observed patterns of detections and re-detections of individual bears to estimate locations of home range centers of bears on the DNA grid and surrounding area therefore providing an initial assessment of distribution of bears that accounts for the layout of sampling sites on the grid.

**Figure 2:**
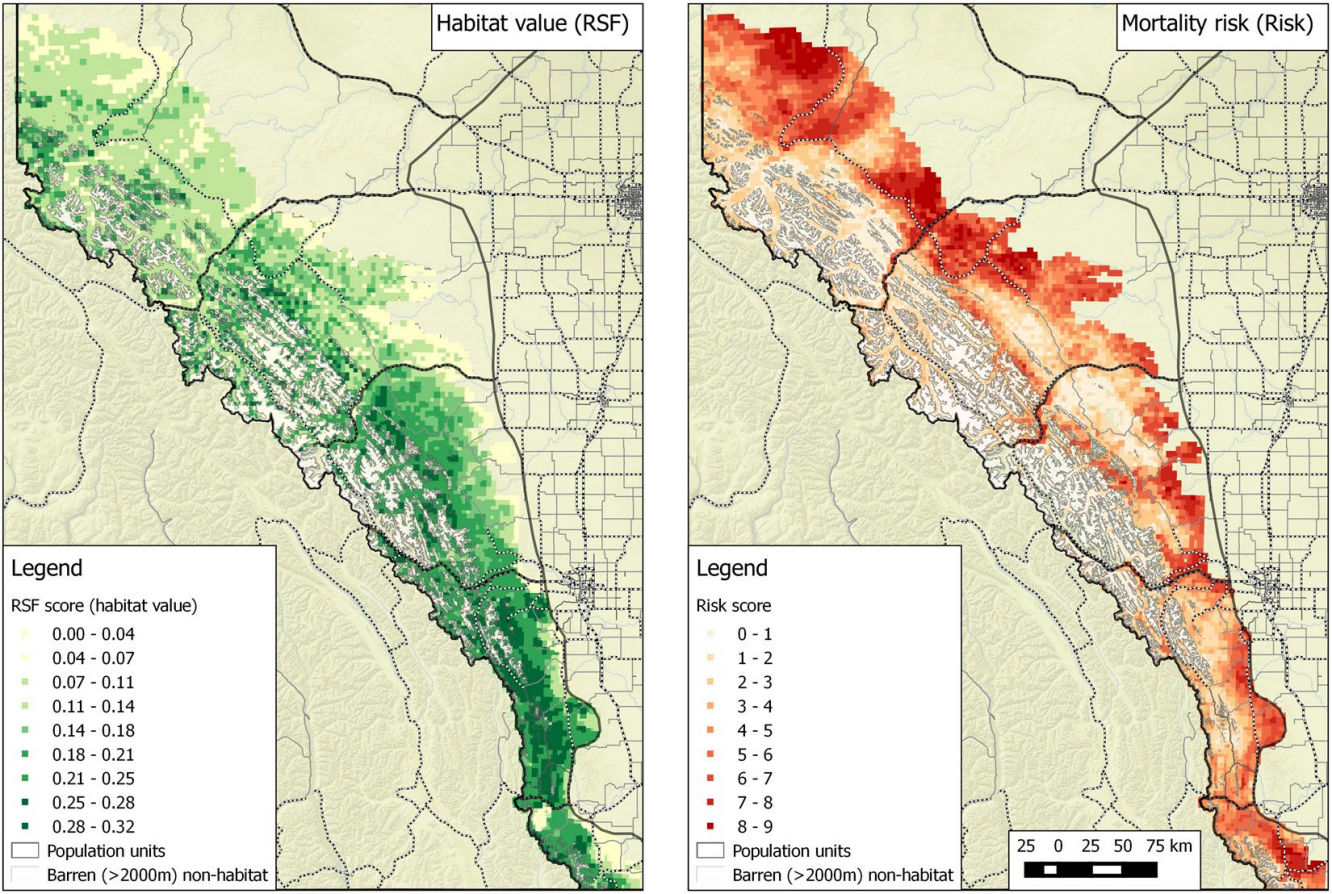
Resource selection function (RSF) scores for grizzly bear habitat (left graph) and mortality risk scores (right graph) used as mask point covariates in the density surface model for analysis of Alberta grizzly bear inventory data (2004–8). In both cases increasing RSF and Risk score suggest increasing habitat value and increasing levels of mortality risk. Map was produced using QGIS software (v2.10.1; qgis.org).

In the second phase of the SECR analysis we fit density surface models that constrained density for each SECR mask point to be a function of habitat and mortality risk covariates. For this analysis, centroids of the SECR habitat mask were populated with RSF habitat values^5^ and Risk values^45^ (Fig. 2) derived from analyses of radio collared bear data. Underlying density models were tested with single RSF or Risk terms, additive RSF and Risk terms, and interactions of RSF and Risk terms. These models tested hypotheses of whether habitat value (RSF), mortality risk (Risk), or an interaction of mortality risk and habitat most resulted in local changes in density. The same suite of models was used for each BMA therefore allowing comparison of the relative support, as reflected by AIC_c_ weights, of each density surface covariate. RSF and Risk was summarized within 1.75 km buffers around each SECR mask point therefore measuring variation in covariates at 3.5 km point spacing. Density surface models were then tested for relative fit compared to the most supported baseline detection models. Density estimates were produced from the most supported models for each sex and year combination that assumed uniform density across the DNA grid and for density surface models.

Of conservation interest was how predicted densities from density surface models and activity centers related to areas of habitat value and relative risk. To explore this, we classified SECR mask centroids with high RSF scores (above the median score) and low mortality risk values as “safe harbor” (source-like) areas, whereas areas of high RSF and high risk centroids were classified as attractive sinks^5^. We then estimated mean density for mask centroids in each category for each BMA to assess the relative importance of safe harbor and attractive sink areas. In addition, we estimated the relative abundance for each RSF/Risk category as the product of mean density and the total mask area to provide a measure of the relative number of bears in each category.

All inferences from density surface models were restricted to the sampled DNA grid areas, as opposed to peripheral mask areas that were not sampled. RSF and Risk map coverage was restricted to Alberta BMAs and therefore some BMA masks (Units 2, 5, and 6) which extended into British Columbia or into areas east of BMAs in Alberta had missing values. For these mask points, RSF and Risk for the nearest non-missing mask point was used. The effect of this interpolation was minimal given that inference was restricted to grid areas which had full coverage of RSF and Risk variables. We also estimated home range centers for bears detected in each BMA to assess what proportion potentially had home range centers in British Columbia.

Analyses were conducted in the program *secr*^35^ in program *R*^48^. Program *secr* uses a maximum likelihood approach to estimate model parameters^33,49^. The closed model and radio telemetry analyses were conducted using program MARK^41^. Quantum GIS (QGIS)^50^ was used for GIS analyses and production of maps with background layers obtained from geogratis.gc.ca. Data were graphically summarized using *ggplot2*^51^ in program R.

### Data availability statement

Data used in this analysis are not available publicly due to conservation concerns of detailing exact locations of collared bears or DNA sites where bears were detected given their threatened status. Data are available with restrictions from the authors of this manuscript.

## Results

DNA results are summarized as the number of bears detected in DNA hair snag sites which varied from 32 in BMA 6 to 269 in BMA 2 (Table 1) with varying levels of sampling efficiency based on the proportion of genotyped bears that were detected in more than 1 sampling occasion. Distribution of bear detections on sample grids was uneven with most detections occurring on the western parts of each BMA with less detections in eastern areas (Fig. 1).

### Spatially explicit analysis of detection and movement

**Figure 3:**
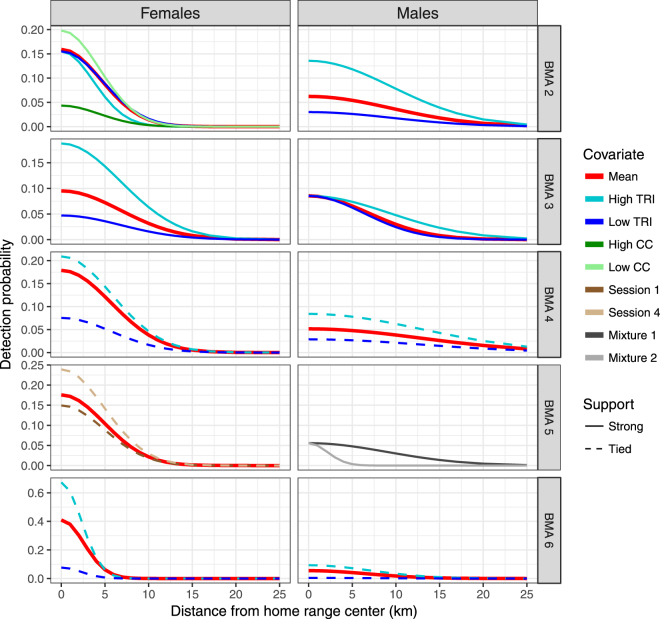
Spatially explicit detection functions for male and female bears as a function of BMA for grizzly bears in Alberta for analysis of Alberta grizzly bear inventory data (2004–8). Note the different scales on the x and y axes. Detection functions are given for non-covariate models (Mean) and as a function of site covariates for g_0_ and σ. Relative support of covariates is delineated by line type. Strong support indicates that covariate models had AICc scores of greater than 2 units than constant models whereas tied support indicates that covariate model AIC_c_ scores were greater than constant models by less than 2 AIC_c_ units. See Appendix S1 for full details of this analysis.

Model selection results for each BMA are summarized in Table 2 based on the most supported SECR models (details of each analysis provided in Appendix S1). The shape of SECR detection functions varied by sex and BMA (red lines in Fig. 3). Females had higher detection rates at the home range center than males with detection at distances out to 10–15 km. In contrast, males displayed lower overall detection rates than females at their home range center and much broader range of detection out to 30 to 40 km from their home range center. Detection functions were similar for most BMAs. One exception was a higher detection rate at the home range center for females in BMA 6 which had lower overall scale of movement.

**Table 2:** Summary of model selection results for each bear management area for analysis of Alberta grizzly bear inventory data (2004–8).

BMA	Detection (g_0_)/scale of movement (σ)				Density surface predictor (AIC_c_ weight)	
	Females		Males		Females	Males
	g_0_	σ	g_0_	σ		
2	CC (−)	TRI (−)	TRI (+)		RSF*Risk (0.57) RSF (0.32)	RSF*Risk (0.48) RSF (0.35) RSF + Risk(0.17)
3	TRI (+)			TRI (+) Trend (−)	RSF (0.75)	RSF (0.98)
4	TRI (~ +)^A^	Trend (+)	TRI (+)^A^		RSF (0.86)	RSF (0.70)
5	Trend^A^ (~ +)			heterogeneity	Risk (0.65)	Risk (0.44) RSF + Risk (0.41)
6	TRI (+)^A^		TRI (+)^A^		Risk (0.81)	Risk (0.48) Constant (0.37)

^A^These covariates were tied for support with AIC_c_ scores that were greater than the constant model by less than 2 AIC_c_ units.

SECR site covariates include CC (Canopy closure), TRI (terrain ruggedness) and Trend (linear trend over sampling sessions). Heterogeneity refers to a mixture model with 2 classes. Supported density surface models (∆AIC_c_ < 2) along with AIC_c_ weights are also shown for each BMA and sex of bear. Detailed model selection results, as well as SECR parameters for each model, are given in Appendix S1.

Analysis of covariates influencing detection (Table 2 and Fig. 3) revealed that terrain ruggedness (*TRI)* positively influenced detection of bears at their home range center (*g*_0_) for both males and females in BMAs 2, 3 and 6 (Table 2). Canopy cover negatively affected detection at home range center for females in BMA 2. Scale of movement (σ) was negatively affected by terrain ruggedness for females in BMA 2, but positively affected for males in BMA 3. In all cases, the site covariate models were more supported than bear-specific heterogeneity and behavioural response models except for males in BMA 5 where a mixture model with undefined heterogeneity in σ was most supported. In BMAs 2 and 3 site covariate models were strongly supported with weaker support in BMAs 4, 5, and 6 as indicated by confidence limits of beta parameters (Appendix S1) and AIC_c_ scores of covariate models being less than 2 units greater than constant models (suggesting models were tied for support). We note that covariates for g_0_ and σ were still supported after the addition of density covariates in phase 2 of the analyses, except for males in BMA 6 where site covariate and null models were tied for support when density covariates were used presumably due to sparse data (Appendix S1).

The second phase of the analysis used the most supported base detection models developed in phase 1 to explore density variation on the sampling grid. In all BMAs, density surface models with RSF, Risk, or combinations of RSF and Risk were more supported than models that assumed homogenous density. More than one density surface model was supported in BMA’s 2, 5, and 6 suggesting that multiple factors were influencing density. In BMA 2, which also had the highest bear densities, models with RSF and Risk were most supported. In BMAs 3 and 4 that are south of BMA 2, models with RSF as the main predictor were most supported. In the furthest south BMAs of 5 and 6, Risk was the most supported predictor of density. Model certainty was higher for females than males in all BMAs except BMA 3, as indicated by higher weights for the most supported models which indicated definitive support for a single model predicting density.

### Closed models and telemetry

**Figure 4:**
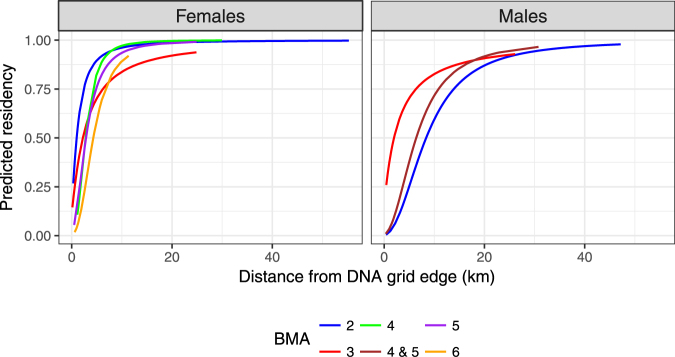
Predicted residency times for radio collared bears as a function of mean location on the DNA sample grid to the outer edge of the grid from closed model/telemetry analysis for analysis of Alberta grizzly bear inventory data (2004–8). See Appendix S2 for full details of this analysis.

Low sample sizes of collared males for some BMAs (Table 1) prevented modelling of residency as a function of distance from edge for each BMA. To offset this issue, radio locations were pooled for BMAs 4 and 5 with residency set at mean levels for BMA 6 (Appendix S2). Predicted residency of grizzly bears varied by sex and by BMA with estimated female residency close to 1 for bears with distances from grid edges that were of >15 km. In contrast, male residency did not approach 1 for distances of up to 30 km (Fig. 4).

### Comparison of closed model and SECR density estimates

**Figure 5:**
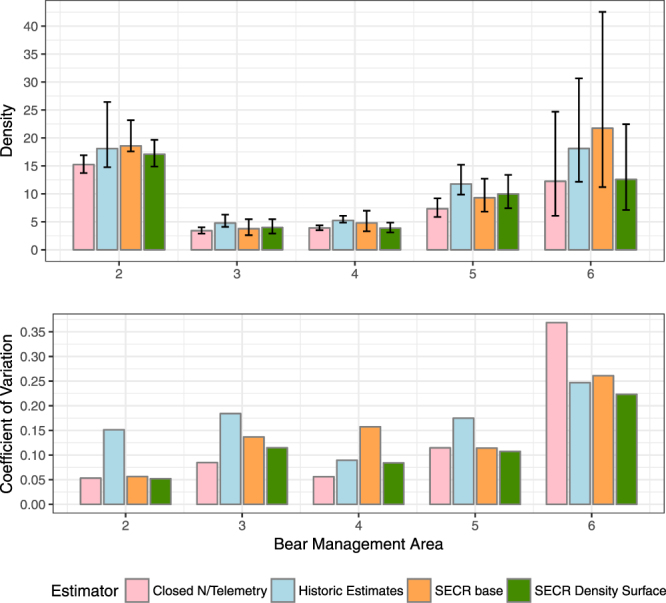
Comparison of density estimates (bears per 1000 km^2^) from closed models and different formulations of SECR models for analysis of Alberta grizzly bear inventory data (2004–8). Density estimates are based on the full grid that include areas of non-habitat. A full listing of estimates is given in Appendices 1 and 2.

Spatially explicit methods based on density surface modelling were lower than base SECR models and historic estimates that assumed similar density for the overall grid areas (Fig. 5). Estimates from the closed model/telemetry estimator were most similar to the SECR density surface model. In all cases, confidence limits overlapped each other when comparing all methods. However, precision was higher for SECR methods compared to estimates from historically used methods and were similar to those of the Closed N/Telemetry model. Notably, precision was poor for all methods in BMA 6 due to lower detections of bears and poor sampling efficiency (Table 1 and Appendices S1 and S2). Robust comparisons were therefore not always possible.

### Spatial variation on sample grid

#### Home range centers

**Figure 6:**
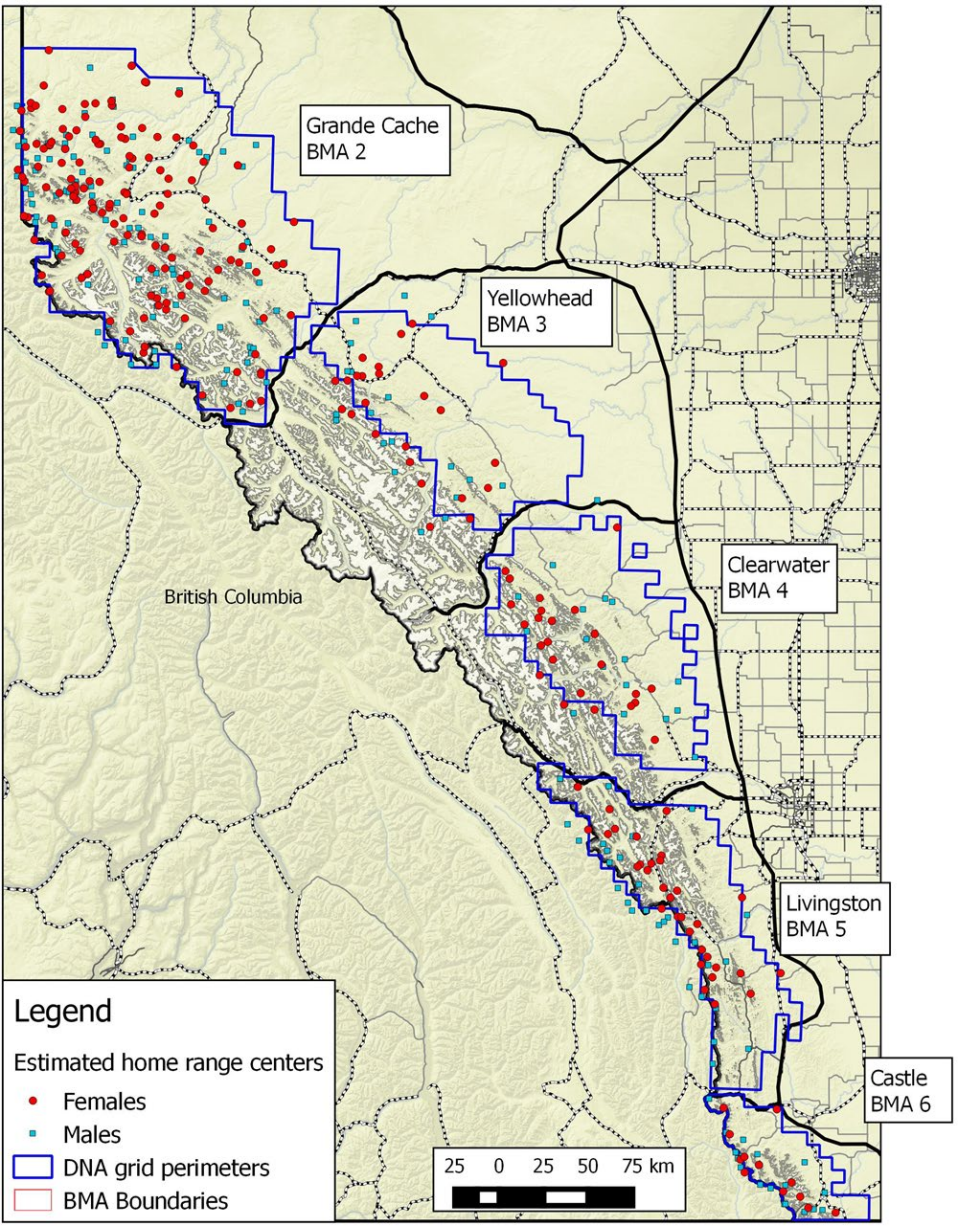
Predicted home range centers from most supported detection models of Alberta grizzly bear inventory data (2004–8). The BMA and year it was sampled is labeled in each map. Map was produced using QGIS software (v2.10.1; qgis.org).

We estimated the home range center locations for bears on the sampling grids to assess relative distribution of bears (Fig. 6) revealing the highest densities of bears in the western portions of most sampling grids. From this, we estimated the relative frequency of bears that were detected on Alberta grids that had home range centers in British Columbia for grids (BMA 2, 5, and 6) bordering British Columbia. Of the 258 female bears detected, 14 (5.4%) had home range centers in British Columbia. Of the 197 male bears detected, 32 (16.2%) had home range centers in British Columbia. The highest proportion of bears with home ranges in British Columbia was BMA 5 (14 of 33 males (42.2%) and 5 of 35 females (14.2%)) and BMA 6 (5 of 19 males (26.3%) and 2 of 13 females (15.3%)). Sampling grids in BMAs 3 and 4 were not adjacent to the British Columbia border (Fig. 1) with a small proportion of bears in BMA 2 having home range centers in BC (13 of 108 (12.0%) males and 7 of 161 (4.3%)).

#### Density surface models

**Figure 7:**
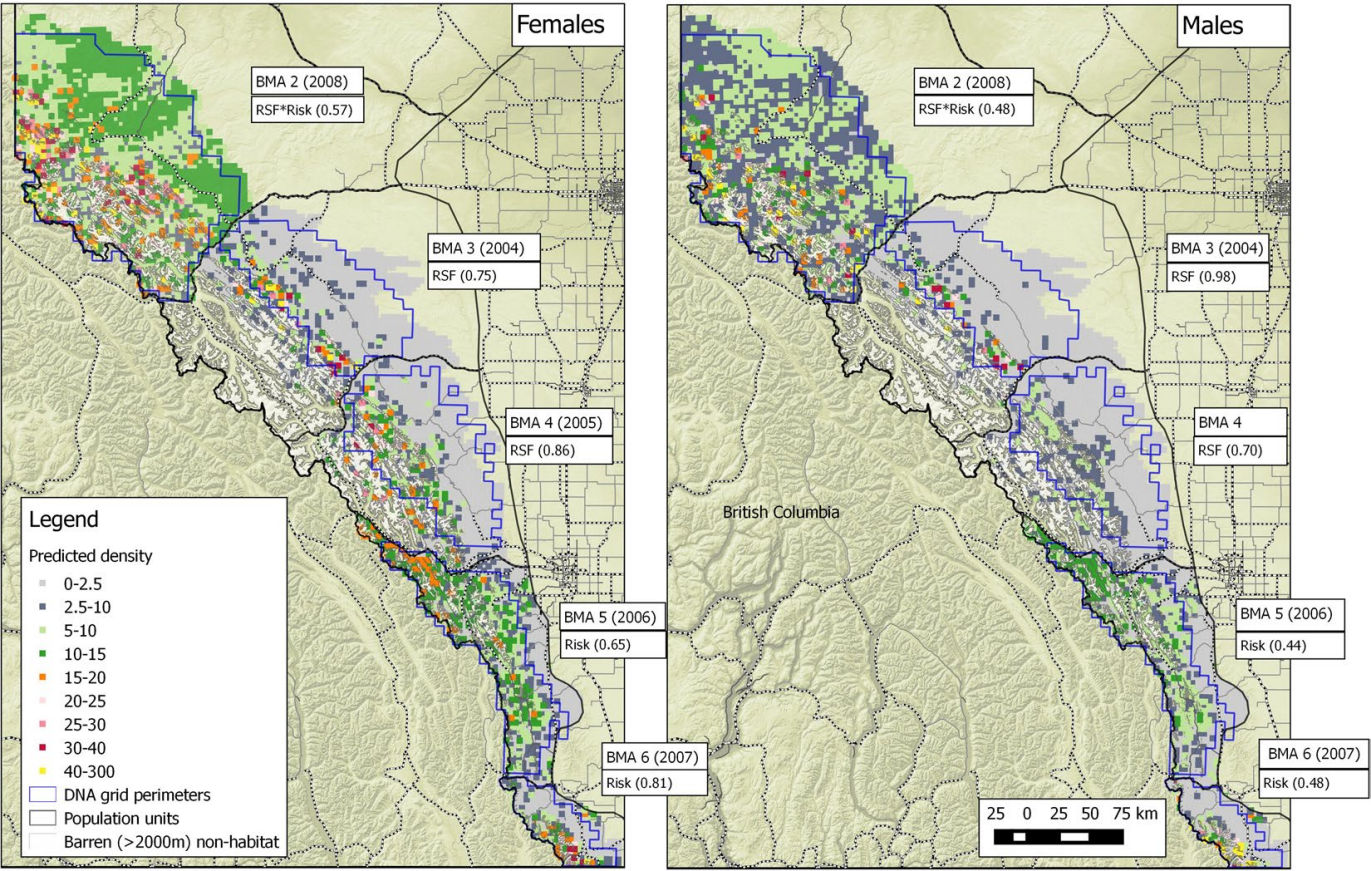
Predicted densities (per 1000 km^2^) of female (left) and male (right) grizzly bears for each bear management area (2004–8). The most supported density surface model used for predictions are shown next to each BMA with its AIC_c_ weight. See Table 2 and Appendix S1 for further details on density surface models supported for each BMA. Map was produced using QGIS software (v2.10.1; qgis.org).

Density surface models were more supported in all BMAs than models that assumed homogenous density, although the overall AIC_c_ support varied by sex and BMA (Table 2, and Appendix S1). Densities from the most supported density surface models suggested that the highest densities were most often in the western parts of BMAs along the mountains except for BMA 2 that had more moderate densities throughout the area (Fig. 7). In general, predictions from density surface models corresponded to predicted home range center locations (Fig. 6) for male and female grizzly bears. As demonstrated in Fig. 7, it is possible to extrapolate predicted density beyond sampled grid boundaries under the assumption that the relationship between predictors and density is similar in extrapolated areas to sampled areas. We restricted all estimates to only areas within sampled grids.

**Figure 8:**
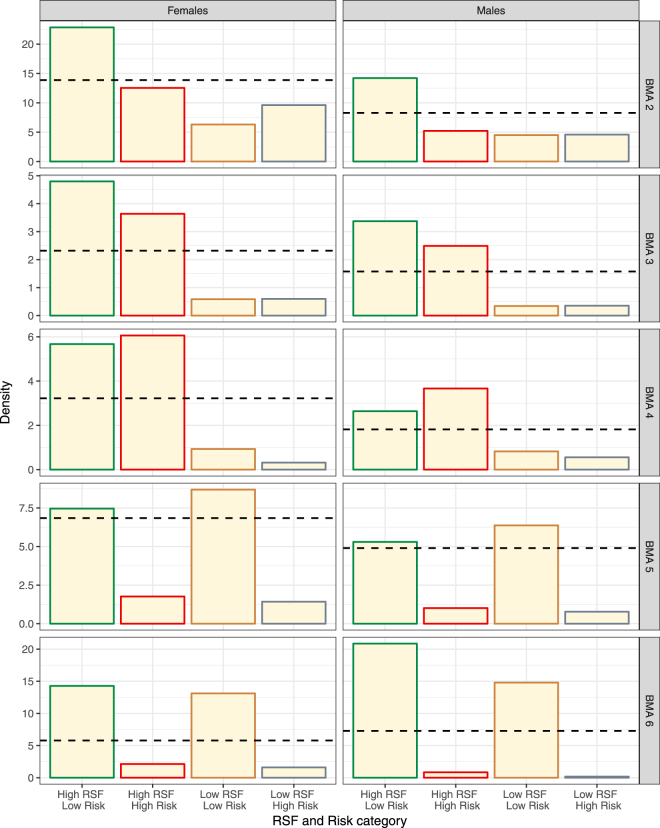
Predicted mean density of female (left) and male (right) bears as a function of RSF and Risk categories by Bear Management area (graph row as labelled in right side of graph) based on density surface models. RSF and Risk areas of “safe harbor” (high RSF and low Risk) are outlined in green, whereas areas of “attractive sink” are outlined in red (high RSF and low Risk). A dashed line indicates the mean density estimate for the given BMA. Areas of low habitat value and risk are outlined in brown and areas of high risk and low habitat value are outline in grey.

There was a general trend of RSF or RSF and Risk density surface models being more supported in the north (BMAS 2, 3, and 4) and Risk models being more supported in the south (BMAs 5 and 6) (Table 2). This general trend, as well as the relative differences between density surface models and activity centers for each BMA, was summarized in terms of mean density as a function of RSF and Risk categories (Fig. 8). The hashed line in Fig. 8 illustrates the mean density for each BMA and therefore deviations from the mean density line for each RSF/Risk category indicates relative selection or aversion to each category.

In general, density was positively related to RSF and inversely related to Risk (Fig. 8). Predicted bear densities were higher in the high RSF/low Risk areas for a density surface for all sexes and BMAs with the exception of BMA 4 in which higher densities were predicted in the high-RSF and high-Risk areas. In BMA 6, a strong gradient in density was indicated by both density surface and activity centers with low densities in areas of high Risk regardless of RSF value. These results further suggest that risk of mortality is the principal limiting factor of bear density in southern areas with Risk playing a lesser role in northern BMAs.

The overall support for Risk as a predictor in BMA 5 was low for females (Table 2) and therefore observed densities, as indicated by activity centers was closer to mean values (as indicated by the dashed line) than predicted by density surface models using Risk as a predictor. This may have been due to a larger proportion of bears with home range centers in British Columbia where sampling did not occur, and covariate coverage limited to Alberta thereby reducing the certainty for the overall distribution of bears in BMA 5.

**Figure 9:**
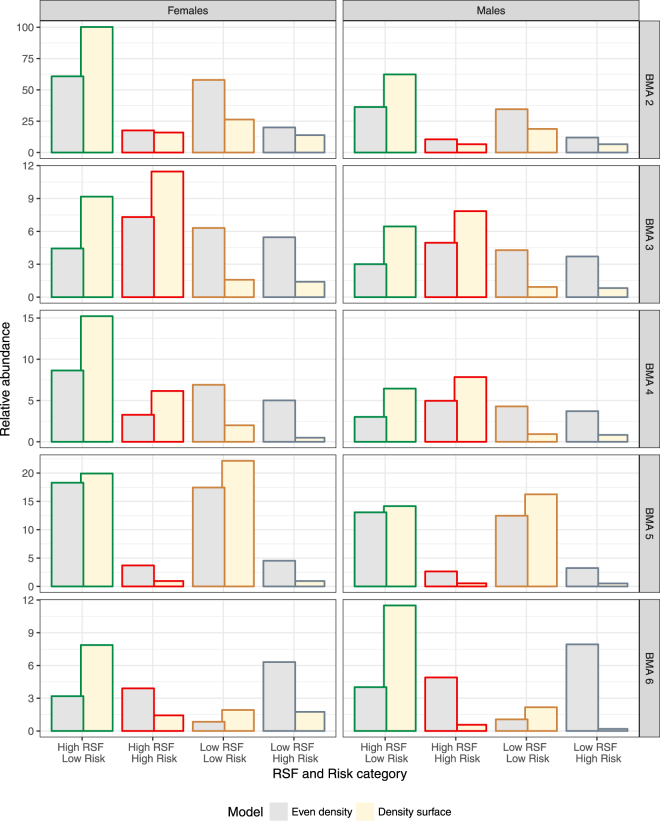
Predicted relative abundance of female (left) and male (right) bears as a function of RSF and Risk categories by Bear Management area (graph row as labelled in right side of graph) based on density surface models and activity centers. Density surface predictions are based on the most supported density surface for each BMA (Table 2). RSF and Risk areas of “safe harbor” (high RSF and low risk) are outlined in green whereas areas of “attractive sink” are outlined in red (high RSF and low risk). Areas of low habitat value and risk are outlined in brown and areas of high risk and low habitat value are outline in grey.

In terms of conservation, the other factor that needs to be considered is the area of each RSF and Risk category in each BMA since this will affect the number of bears influenced by RSF and Risk categories per BMA. We multiplied the estimated densities in Fig. 8 by the area of each RSF and Risk category to estimate the relative abundance of bears (Fig. 9). We also added a relative abundance estimate from a constant density base model which corresponded to a situation in which abundance was assumed to be directly proportional to the amount of area of each category. From this it can be seen that safe harbor (High RSF/Low Risk) habitats occupied the most area (as indicated by estimates of abundance assuming even density) in BMAs 2, 4, and 5, whereas attractive sink habitats (High RSF/High Risk) occupied the most area in BMAs 3 and 6.

## Discussion

Our analyses demonstrate that spatially explicit methods provide robust estimates of grizzly bear density, as well as inferences about landscape factors influencing density. This extends the amount of inference from mark-recapture data beyond simple point estimates of abundance or density. An important advantage of SECR is that radio collared bears are not required for estimates of density which makes the method more applicable to more areas. The spatial sample of bears is also much larger and more representative than the smaller sample of radio collared bears on most sample grids. Therefore, it could be argued that SECR will provide a better estimate of effective sampling area than radio telemetry and traditional mark-recapture studies which are normally represented as having smaller sample sizes of radio-collared bears^52^. The second advantage of spatially-explicit methods is that it allows modelling of variation in density, thus expanding the scope of inference from a single estimate of density or abundance. For example, it is possible to obtain estimates of smaller regions of the sampling grid and to assess factors influencing distribution at larger scales. This also allows management actions in specific areas to target actions for recovery with trend monitoring occurring within these smaller regions thereby reducing sampling costs.

The general agreement between SECR and closed telemetry methods (Fig. 5) suggests that in terms of overall precision there is little difference between methods, although we note that bias cannot be truly inferred in this comparison given that the true values are not known. The similarity in approaches can also be observed by the general range of movements indicated by SECR detection functions (Fig. 3) and telemetry-based residency curves (Fig. 4). In both cases, each curve indicates that the scale of movement of a bear on a grid, and thus detectable with DNA hair-snagging, is approximately 15 and 30 kilometers for females and males, respectively. The main challenge of using a telemetry-based estimator is obtaining adequate sample sizes of collared bears to fully cover the study area. We used data from previous years for the same season as DNA sampling to estimate residency for each of the bear management areas. However, sample sizes were still low for some areas such as BMA 6. In addition, the distribution of collared bears will be influenced by areas of higher access where it is easier to capture and collar bears and the amount of open habitat where aerial capture is possible. Therefore, the distribution of collared bears may be unrepresentative of the overall population. Using distance from edge as a covariate reduces bias if it can be assumed that movements of collared bears are similar to non-collared bears across the sampling grid. The main challenge is that collared bears will be represented mainly by adults, as opposed to all age classes of bears. In contrast, movements estimated from spatially-explicit methods like SECR provide a more representative sample of bears if there are adequate numbers of spatial recaptures.

Use of covariates such as terrain ruggedness and canopy closure demonstrated that terrain and land cover affects both detection of bears and scale of movement as illustrated in previous studies^53^. Bears are easier to detect in valleys with higher terrain ruggedness values, as well as lower canopy cover. Terrain ruggedness also affects scale of movement with reduced movements in areas of higher terrain ruggedness. The use of site covariates provides a useful alternative to mixture heterogeneity models as a means of describing variation in detection probability. However, it should be noted that heterogeneity in site detection probabilities does not necessarily cause a negative bias in estimates (as does heterogeneity of bear detection probabilities). For example, many some than constant models (Appendix S1). In this case, the detection rate of bears in the mountains was higher. Since this was where many bears were detected, the net result was a lower estimate for the entire grid.

Spatially explicit mark-recapture models do make assumptions about the movements and home range shape of bears that should be considered when evaluating estimates. The first assumption is that home ranges are circular in shape with a central tendency so that movement can be described using a detection function. Simulation studies^44^ suggest that negative bias is possible if home range size is elongated. However, recent studies contest this claim (Murray Efford, University of Otago, NZ, Per. Comm). A second assumption is that home range centers are stationary during sampling with minimal transient movement of individuals. This assumption allows the population to be defined in time and space and is central to any estimation method. The general findings from simulation studies is that density estimates will be reasonably robust to transient movement, although estimates of the scale of movement may be biased^54^.

We note that a fundamental assumption of our methods is that bears that encounter hair snag sites had a non-zero probability of detection. By modelling trap layout, spatially explicit methods confront potential heterogeneity caused by trap layout which may cause some bears to have low detection probabilities^55^. In this case, the assumption of the spatially explicit methods is that the landscape is sampled in a representative fashion so that all areas within the defined unit of inference have a probability of being sampled^14^. Some authors have used rub trees to sample bears^56–58^ which could cause different estimates given differential vulnerability of male and female bears to rub tree sampling compared to hair snag sampling, especially if female bears have 0 detection probability for rub trees during specific sampling periods^59^. We also note that our inference applies to the spring season when sampling occurred. Sampling that occurs into the summer berry season and fall may increase estimates and potential patterns of distribution given changes in bear movements during these seasons^56^, although another study^13^ did not find changes in detectability between spring and summer periods for hair snags. A recent study that utilized collared bears to estimated rub tree detection probabilities documented temporal sex-specific variation in rub tree detection probabilities over seasons^60^. We also note that rub trees with spray-on scent lures are different than hair snags that utilize a large amount of liquid lure and do not rely on bear rubbing behavior for hair snag detections, as suggested by some studies^57^.

The density surface modelling approach used in this study provides a spatial representation of estimates allowing robust estimates of sub-areas of each of the DNA grids (Fig. 7). Density surface model estimates were lower than those that assumed homogenous density, but the overall differences were minor. Simulation studies suggest that SECR methods should be robust to uneven densities within sample grids^14,15^, however, other studies have suggested that density surface models do display lower estimates than constant density models^61^. Many of the Alberta DNA grids have a strong gradient of density from higher density in the higher elevation mountainous areas to the west and lower densities in the foothills and plains to the east with home range centers often occurring on the west edge of sample grids (Fig. 6). In this case, the density surface model may provide a better spatial representation of density which leads to a different estimate. Regardless, we suggest that interpretation of variation in density within sampling grid areas provides more insight into status of bear populations than a single estimate of density which was produced using historical methods. This information can be useful for targeted management strategies for both habitat improvement and mortality risk reduction.

Previous methods that have used DNA data to assess distribution of bears have modelled variation in density using the relative number of detections at hair snag sites with inference based upon assumed scales of movement around the DNA sites^2,12,62,63^. This approach often assumes similarity in detection probabilities at sites and similar scales of movement relative to sites. The results of this study suggest that detection probabilities at sites, as well as scales of movement, vary by sex, management unit, and habitat factors such as canopy cover and terrain ruggedness (Fig. 3). Unlike RSF models based on frequencies of detections at hair snag sites, spatially explicit methods that model density use an evenly-spaced mask grid with the response variable being location of home range centers, rather than the number of detections at a hair snag site. This approach should therefore be more robust to sampling variation between projects, as well as the effect of placement of DNA hair snag sites in high RSF habitat. However, we also note that detection functions for male and female bears (Fig. 3) suggest that bears display non-zero detection across scales larger than the 7 × 7 km grid cells used for sampling and therefore placement of sites in micro-habitats within cells should not greatly influence results especially since sites were moved between sessions in all projects. It would also be possible to use raw satellite imagery and observed road densities to generate land cover data with which to model density and Risk rather than RSF and Risk score. This approach could provide a useful comparison to determine if RSF’s derived from GPS collared bear data and land cover mapping is a better predictor than other habitat features. An example of this approach is a recent study in British Columbia which used spatially explicit methods to estimate threshold road densities needed for population recovery of grizzly bears^64^.

We suggest that the density surface approach, combined with graphical representation of estimates of density and relative abundance estimates based on supported density surface models, provides a better method for interpreting and understanding factors limiting grizzly bear populations in bear management areas in Alberta (Figs 8 and 9). In some BMAs, such as BMA 2 and 5, results suggest that both RSF and Risk influence bear density given support for multiple density surface models. We speculate that the influence of each factor will depend on the density of bears on sampling grids relative to the carrying capacity and historical patterns of mortality, as well as the statistical power to discern patterns in density.

The actual distribution of Risk and habitat value (RSF) varies greatly for each BMA (Figs 2 and 8). From previous research^5^, the high RSF and low Risk areas can be conceptualized as safe harbor (source-like) areas, whereas the High RSF and High Risk are attractive sinks. The low RSF habitats are buffer areas that might be colonized as densities of bears reach carrying capacity. In this context, BMA 2 has a greater proportion of source compared to sink habitats with positive selection for the source areas as indicated by higher abundances from density surface and activity center models compare with mean density predictions. In contrast, BMA 3 to the south has higher proportions of both source and sink habitats with positive selection for both types suggesting an active source-sink dynamic in the bear management areas as indicated by both density surface and activity center models. BMA 4 has a higher proportion of safe harbor habitat; however, selection for sink habitats is also suggested as indicated by higher densities in the high Risk-high RSF category. In BMA 5, much of the area is safe harbor habitat with similar predicted abundances assuming equal density and home range centers and density surface models. In BMA 6, safe harbor areas, such as Waterton National Park, have higher abundances with negative selection for other categories as also suggested by support of Risk as a predictor of density on the sampling grid. The relatively large amount of sink habitat in BMAs 3 and 6 suggests that conservation measures here need to focus on management of bear mortality if populations are to recover and colonize higher risk habitats. Sink habitat in BMA 3 is created by roaded habitat in forestry areas^65^ whereas sink habitat in BMA 6 is created by roads in combination with agricultural areas^5,56^. In conclusion, these results demonstrate that bear abundance is distributed along a source-sink gradient, although the relationship is BMA-specific and depends on the relative availability of source and sink habitats.

The general source-sink dynamic with areas of high-valued habitat created by forestry, agriculture, and mining has been noted in previous studies utilizing radio collared bears^3,6,7,65^. However, these studies have not been able to assess how the status and distribution of bears are affected by source and sink dynamics. A conservation question is whether mortality risk can be managed in BMAs 3 and 6, which have significant attractive sink habitat, to maintain population viability. The SECR approach could be repeated each time an area is sampled or as relative risk of areas is updated to determine the success of conservation measures. For example, if sampling is repeated and conservation measures are employed, then a shift from Risk to RSF as the predictor of density would suggest that conservation measures had been successful. In addition, relative abundance of bears in areas serves as a potential metric for evaluating recovery actions for this threatened species in Alberta. This moves inference from DNA studies beyond simple interpretation of point estimates of abundance and density. We note that SECR methods can be employed using stratified or cluster sampling^14,66,67^ with less dependence on large scale and expensive uniform trap density grid designs therefore making this approach more cost-effective then previous DNA mark-recapture efforts. We see these results and methods as providing important new approaches for long term monitoring of recovery efforts for this threatened species in Alberta.

## Electronic supplementary material

Appendix S1 and S2
