# Supplementary material for: Using spatial mark-recapture for conservation monitoring of grizzly bear populations in Alberta
Source: Sci Rep. 2018 Mar 26;8:5204. doi: 10.1038/s41598-018-23502-3 (PMC5980105; doi:10.1038/s41598-018-23502-3)
Supplement: Supplementary file 1 — Appendix S1 and S2 [file 41598_2018_23502_MOESM1_ESM.docx]

**An assessment of spatial mark-recapture as a conservation tool for monitoring grizzly bear populations in Alberta**

**John Boulanger^1,*^, Scott E. Nielsen^2+^, and Gord Stenhouse^3+^**

^1^ Integrated Ecological Research, 924 Innes St., Nelson, BC V1L 5T2 Canada

^2^ Department of Renewable Resources, University of Alberta, 751 General Services Building, Edmonton, AB T6G 2H1, Canada

^3^ fRI Research 1176 Switzer Drive, Hinton, Alberta, T7V 1V3, Canada

*** boulange@ecological.bc.ca

# Appendix S1

# Information on spatially explicit analyses of grizzly bears for each Alberta Bear Management Area

In this document we detail the analysis of each BMA area as summarized in the full manuscript text. We note that details about each area and detailed summaries of the data sets are given in individual reports that are available online (Table 1).

Table 1: Summary of grid areas for Alberta DNA mark-recapture inventory projects by bear management area (BMA) and year sampling occurred. The citations are available online from the government of Alberta (<http://aep.alberta.ca/fish-wildlife/wildlife-management/grizzly-bear-recovery-plan/>) using the links in the literature cited section.

|  |  | Area of DNA grid (km^2^) | |  |  |
| --- | --- | --- | --- | --- | --- |
| BMA | Year | Total | Habitat | % Habitat | Source of information |
| 2 | 2008 | 19,502 | 17,133 | 87.7% | Inventory report^1^ |
| 3 | 2004 | 8,820 | 8,514 | 96.5% | Inventory report^2^ |
| 4 | 2005 | 9,016 | 7,820 | 86.7% | Inventory report^3^ |
| 5 | 2006 | 8,134 | 6,868 | 84.4% | Inventory report^4^ |
| 6 | 2007 | 2,827 | 2,584 | 91.4% | Inventory report^5^ |

To estimate the size of the mask, relative to study area size, needed to minimize bias in density estimates, the *esa.plot* function in program *secr*^6^ was run for sex-specific *g_0_* and σ models for each BMA. The *esa.plot* shows the effect of increasing mask size on estimates of the effective sampling area of the DNA grid. The appropriate mask size is then determined when estimates of effective sampling area do not depend on the size of the mask. Spacing of SECR mask centroids were 3.5 km on all DNA grids. Sensitivity analyses suggested that this spacing optimized computation time with minimal changes in estimates compared to tighter spacing of mask centroids.

As described in the main paper, the modelling process started with development of baseline models to describe variation in detection probabilities at home range center (g_0_) and scale of movement (σ). The main covariates tested for each BMA are summarized in Table 2. Site covariates pertain to each hair snag site and include habitat covariates summarized at home range (10 km buffer) or site (1.69 km buffer) scales. Also tested was the effect of fixed versus moved sites which mainly pertained to Units 2 and 3 where a proportion of sites were not moved between sessions. For other BMA’s all sites were moved between sessions except for a small proportion of fixed “transect” sites which extended into the eastern boundaries of study areas. For fixed sites learned response where a site’s detection probability changes after initial detection, as well as transient response (the detection probability changes based on last session where detection occurred). Bear covariates pertain to individuals detected during sampling with temporal trends (t and T), behavioural response (change in detection after initial detection) and undefined heterogeneity models being considered. Continuous site covariates were converted to ordinal classes (6-9 classes) for analyses to optimize the memory required for each model run. Predicted home range centers for the most supported detection models were generated using the *fxi.contour* command in *secr* with the *fitmode* option set to true.

Table 2: Site habitat and sampling covariates used to describe scale of movement and detection of bears

| Covariate | Description |
| --- | --- |
| Site covariates |  |
| TRI | Terrain ruggedness index ^7^ |
| CC | Percent canopy cover |
| dstream | Log of distance to nearest stream |
| fix | Whether a site was moved or fixed during sampling (BMA 2 and 3 only) |
| Fix*K | Site transient response for fixed sites (BMA 2 and 3 only) |
| Fix*k | Site learned response for fixed sites (BMA 2 and 3 only) |
| Bear covariates |  |
| h_2_ | Mixture model to describe heterogeneity variation^8^ |
| t | Session-specific variation in g_0_ or σ |
| T | Linear trends in g_0_ or σ |
| b | Behavioural response (change in g_0_ after initial detection) |

Once the baseline detection model was density surface models were run with results being compared using Information theoretic methods. AIC_c_ weights were used to evaluate the relative strength of support for each model. The suite of 6 models considered for each analysis were the most supported detection covariate model with constant density, the 4 density models (RSF, Risk, RSF*Risk, RSF+Risk) and model with all parameters constant. Using the same suite of models for each analysis allowed the comparison of relative model weights for density surface models across BMAs.

As a separate analysis, a model with the most supported density covariates but with constant detection models was evaluated to verify that detection parameters were still supported when density surface variation was included in the model. Model selection AIC_c_ scores from the density surface/constant detection model was not included in the main analysis to avoid confounding model weights associated with support of density surface covariates (reported in Table 2 and Figure 7 in the main manuscript) with those pertaining to detection parameter covariates. However, these results are reported in this supplementary material section.

The strength of density surface predictors was evaluated graphically using predictions for each mask centroid as a function of the most supported density surface model covariates. The *predictDsurface* command in *secr* was used to generate predictions for the most supported density surface model. Abundance and density estimates were derived for the most supported models to assess relative sensitivity of estimates to model parameterization. In addition, historical closed model/telemetry estimates from the original inventory projects (Table 1) were listed and compared to spatially explicit estimates. We note that a full comparison of estimates is given in the main manuscript.

Results are now summarized by sex of bear and BMA. We provide a consolidated table of model parameters at the end of the appendix.

## Grande Cache (BMA 2)

The Grande Cache BMA 2 inventory was conducted in 2008^1^. This inventory project sampled the largest area (19,526.4 km^2^) and detected the largest number of bears (Table 3). Cell size varied from 7x7 km in the main grid area to 14X14 km in peripheral areas. Overall, 161 females and 108 males were detected with a high degree of sampling efficiency as indicated by capture frequencies (Table 3).

**Table 3: Summary statistics for the 2008 Alberta Grande Cache Area (Bear Management BMA 2) and Jasper National Park grizzly bear DNA inventory project.**

|  | | **Session** | | | | | | | |  |
| --- | --- | --- | --- | --- | --- | --- | --- | --- | --- | --- |
| **Statistic** | | **1** | | **2** | | **3** | | **4** | **Total** | |
| ***Females*** | |  | |  | |  | |  |  | |
| Animals caught (n_j_) | | 50 | | 62 | | 70 | | 77 | 259 | |
| Newly caught (u_j_) | | 50 | | 41 | | 36 | | 34 | 161 | |
| Frequencies (f_j_) | | 91 | | 46 | | 20 | | 4 | 161 | |
| Total individuals caught (M_j_) | 50 | | 91 | | 127 | | 161 | | 161 | |
| Detections | | 58 | | 71 | | 80 | | 88 | 297 | |
| Detectors visited | | 49 | | 54 | | 65 | | 63 | 231 | |
| Detectors available | | 313 | | 315 | | 314 | | 313 | 1255 | |
| ***Males*** | |  | |  | |  | |  |  | |
| Animals caught (n_j_) | | 37 | | 52 | | 44 | | 48 | 181 | |
| Newly caught (u_j_) | | 37 | | 34 | | 15 | | 22 | 108 | |
| Frequencies (f_j_) | | 56 | | 35 | | 13 | | 4 | 108 | |
| Total individuals caught (M_j_) | 37 | | 71 | | 86 | | 108 | | 108 | |
| Detections | | 52 | | 74 | | 54 | | 61 | 241 | |
| Detectors visited | | 47 | | 56 | | 41 | | 53 | 197 | |
| Detectors available | | 313 | | 315 | | 314 | | 313 | 1255 | |

Initial SECR analysis was used to estimate a buffer width of 13.8 kilometers to minimize bias in density estimates. Therefore, the mask buffer was set to 15 kilometers for both males and females. Mask spacing was varied from 2 to 5 kilometers to determine optimal spacing. Density estimates were similar up to 5 kilometer spacing after which density estimates increased. Therefore, mask centroid spacing was set at 3.5 km for analysis in BMA 2 and other bear management areas

## Females

Female bears were detected throughout the grid areas and exhibited moderate movements relative to the high density of HS sites (Figure 1). Overall, 161 female grizzly bears were detected over 4 sampling sessions with 297 unique detections including within session detections (Figure 1 and Table 3). The mean distance moved between detections was 6.7 km with a root pooled spatial variance (RPSV) of 6.3. The RPSV statistic measures the deviation of detections for an individual from the home range center (as estimated by the mean x and y location) therefore providing an index of overall area where individuals were detected^9^.


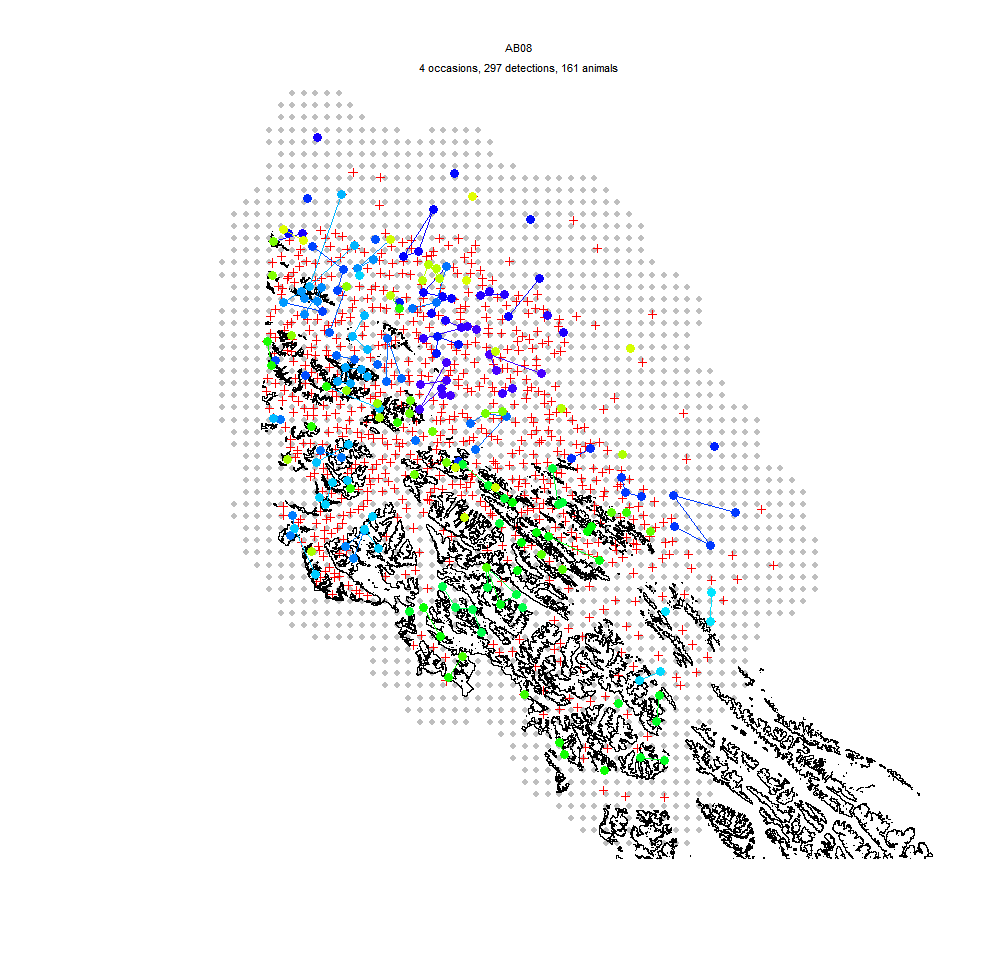


Figure 1: Detections and tracks of female grizzly bears in BMA 2 from repeated detections relative to habitat mask (grey dots), hair snag site locations (red + signs) and barren non-habitat (black polygons with no mask points inside them). The actual sequence of tracks from repeated detections does not necessarily reflect the true path of movements given that the time of within-session detection at hair snag sites is unknown. The cumulative locations of HS sites are displayed which include sites moved each session. Therefore, the actual spatial coverage of HS sites for one session will be overrepresented by this graphic. Map was produced using the *secr* package ^6^ (v 2.9.2; <http://www.otago.ac.nz/density/SECRinR.html>) in program R^10^ (v 3.1.2; [www.r-project.org](http://www.r-project.org) ) plotting functions.

Model selection initially focused on a parsimonious base model for detection and scale of movement. Of models considered, a model with detection at home range centre varying by canopy closure and scale of movement varying with terrain ruggedness index (Model 6, Table 4) was most supported. Density covariates were then tested for support with a model with RSF and Risk and an interaction of RSF risk being most supported (Model 1, Table 4).

A model with the most supported density covariates (RSF +Risk +RSF*Risk) but without the detection covariates (g0(.) σ(.)) was less supported (∆AIC_c_=11.26) verifying that detection covariates were still supported when density variation was included in the SECR model. The results of this model are not included in Table 4 so that the model weights in the primary analysis are solely influenced by the relative support of density surface models without the confounding effect of varying support of detection models.

Table 4: Abridged model selection results for female grizzly bears in the BMA 2 inventory. AIC_c_ = sample size adjusted Akaike Information Criterion , ΔAIC_c_ = the difference in AIC_c_ between the model and the most supported model, AIC_c_ weight = w_i_, K, the number of model parameters and log-likelihood are given. Baseline constant models are shaded for reference with covariate models. The most supported baseline model (of models considered as listed in Table 2) is shown.

| No | Density | Detection | AIC_c_ | ΔAIC_c_ | w_i_ | K | LL |
| --- | --- | --- | --- | --- | --- | --- | --- |
| 1 | RSF +Risk +RSF*Risk | g_0_(CC) σ (TRI) | 2114.3 | 0.00 | 0.57 | 8 | -1048.7 |
| 2 | RSF | g_0_(CC) σ (TRI) | 2115.5 | 1.12 | 0.32 | 6 | -1051.5 |
| 3 | RSF +Risk | g_0_(CC) σ (TRI) | 2117.6 | 3.26 | 0.11 | 7 | -1051.5 |
| 4 | constant | g_0_(CC) σ (TRI) | 2142.7 | 28.38 | 0.00 | 6 | -1065.1 |
| 5 | Risk | g_0_(CC) σ (TRI) | 2143.4 | 29.07 | 0.00 | 5 | -1066.5 |
| 6 | constant | g_0_(.) σ (.) | 2159.8 | 45.48 | 0.00 | 3 | -1076.8 |

A plot of predictions from Model 1 (Table 4) revealed that estimates of density increased with RSF and RISK score, however the effect of increasing RSF score is lessened as RISK score increased as parameterized by the RSF X Risk interaction term in the model (Figure 2).

Risk

0.2

0.4

0.6

0.8

RSF

0.0

0.1

0.2

0.3

0.4

0.5

Density

50

100

150

200

50

100

150

200

Figure 2: Estimated relationship between density RSF and Risk score for female grizzly bears in the BMA 2 inventory as estimated from model 1 (Table 4). Each data point is an estimate of density for a SECR mask centroid.

Estimates of expected (Average N) on sampling grids were reasonably similar for density surface models compared to constant density models as well as closed N/telemetry models run in the original analysis of the data set for the full grid (Table 5). Precision of estimates were higher for spatially explicit models.

Table 5: Estimates of expected size and density for female grizzly bears in the BMA 2 inventory area. Densities (bears/1000 km^2^) are based on total area of the DNA grid as listed in Table 1. The first model and estimates listed for each area is from the most supported SECR model. Closed model/telemetry estimates from original analyses^1^ are given for comparison.

| SECR model | | Expected population size | | | |  |  | Density | |  | |  | |
| --- | --- | --- | --- | --- | --- | --- | --- | --- | --- | --- | --- | --- | --- |
| Density | Detection | $\hat{N}$ | SE | Conf. Int. | | | CV | $\hat{D}$ | SE | Conf. Int. | | |  |
| RSF*Risk | g_0_(CC) σ (TRI) | 214.3 | 14.8 | 187.2 | 245.4 | | 6.9% | 10.99 | 0.76 | 9.60 | 12.58 | |  |
| RSF*Risk | constant | 217.3 | 14.7 | 190.3 | 248.0 | | 6.8% | 11.14 | 0.86 | 11.11 | 14.47 | |  |
| constant | g_0_(CC) σ (TRI) | 228.1 | 15.5 | 199.6 | 260.6 | | 6.8% | 11.70 | 0.91 | 11.65 | 15.21 | |  |
| constant | Closed N/Telemetry | 217.6 | 35.8 | 176.0 | 330.0 | | 16.5% | 11.16 | 2.09 | 10.28 | 19.27 | |  |

### Males

In contrast to females, males were distributed more in mountainous areas with fewer individuals detected in eastern sections of the sampling grid. Males also displayed longer movements especially in the eastern part of the sampling grid (Figure 3). Overall, 108 males were detected with 241 detection events including within session detections (Table 3).


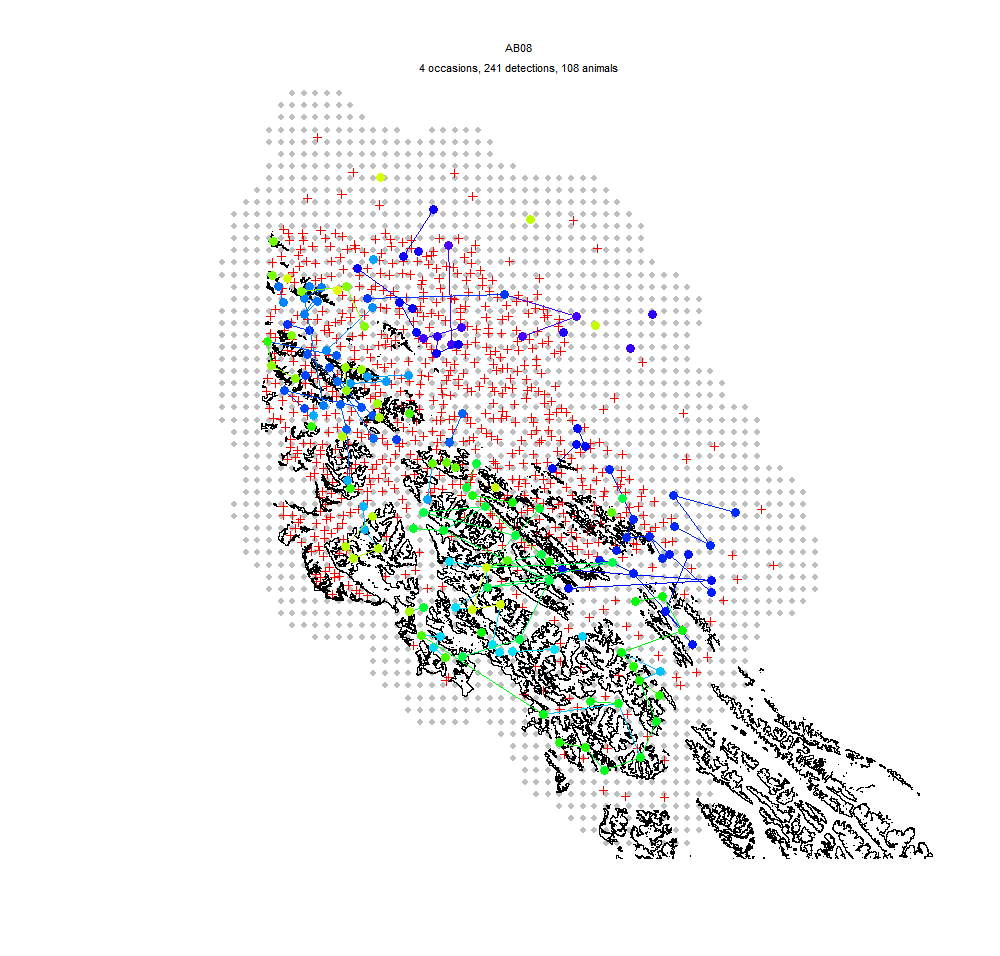


Figure 3: Detections and tracks of male grizzly bears in BMA 2 from repeated detections relative to habitat mask (grey dots), hair snag site locations (red + signs) and barren non-habitat (black polygons with no mask points inside them). The actual sequence of tracks from repeated detections does not necessarily reflect the true path of movements given that the time of within-session detection at hair snag sites is unknown. The cumulative locations of HS sites are displayed which include sites moved each session. Therefore, the actual spatial coverage of HS sites for one session will be overrepresented by this graphic. Map was produced using the *secr* package ^6^ (v 2.9.2; <http://www.otago.ac.nz/density/SECRinR.html>) in program R^10^ (v 3.1.2; [www.r-project.org](http://www.r-project.org) ) plotting functions.

Initial model selection focused on a parsimonious base model to describe movement and detection probabilities. Of models considered, a model with detection probability varying positively with terrain ruggedness index and constant scale of movement was most supported (Model 5 , Table 6). Density surface models were then considered, and of these, a model with density varying with RSF and Risk interactions (Model 1) was most supported.

Table 6: Abriged SECR model selection for Males for the BMA 2 Inventory. AIC_c_ = sample size adjusted Akaike Information Criterion , ΔAIC_c_ = the difference in AIC_c_ between the model and the most supported model, AIC_c_ weight = w_i_, K, the number of model parameters and log-likelihood are given. Baseline constant models are shaded for reference with covariate models. The most supported baseline model (of models considered as listed in Table 2) is shown.

| No | Density | Detection/scale | AIC_c_ | ∆AIC_c_ | w_i_ | K | LL |
| --- | --- | --- | --- | --- | --- | --- | --- |
| 1 | RSF +Risk +RSF*Risk | g_0_(TRI) σ (.) | 2126.47 | 0.00 | 0.48 | 7 | -1055.7 |
| 2 | RSF | g_0_ (TRI) σ (.) | 2126.78 | 0.31 | 0.35 | 5 | -1058.1 |
| 3 | RSF +Risk | g_0_ (TRI) σ (.) | 2127.51 | 1.04 | 0.17 | 6 | -1057.3 |
| 4 | Risk | g_0_ (TRI) σ (.) | 2135.63 | 9.16 | 0.00 | 5 | -1062.5 |
| 5 | constant | g_0_ (TRI) σ (.) | 2136.68 | 10.21 | 0.00 | 4 | -1064.1 |
| 6 | constant | constant | 2157.86 | 31.39 | 0.00 | 3 | -1075.8 |

A model with the most supported density covariates (RSF +Risk +RSF*Risk) but without the detection covariates (g0(.) σ(.)) was less supported (∆AIC_c_=7.13) verifying that detection covariates were still supported when density variation was included in the SECR model.

A plot of predicted density as a function of RSF and Risk (from Model 1 in Table 6) revealed highest densities at low risk and higher RSF with most mask points occurring in moderate RSF and RISK scores (Figure 4).


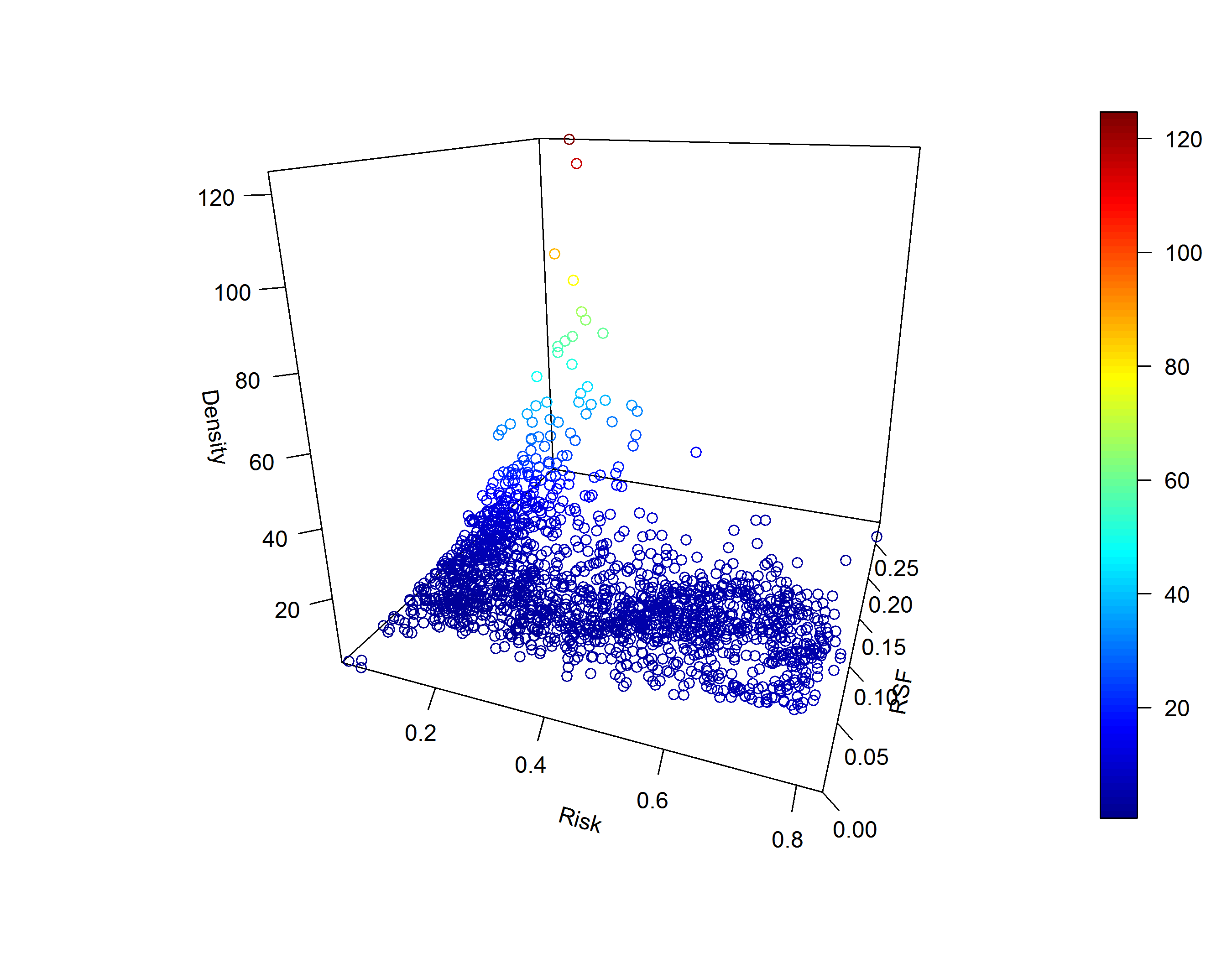


Figure 4: Estimated relationship between density, Risk, and RSF scores from model 1 Table 6.

Estimates of population size on the sampling grid were relatively similar with a slight increase in estimates if density was assumed to be constant (Table 7). Estimates were reasonably close to those derived from closed models and telemetry.

Table 7: Estimates of expected population size and density for male grizzly bears. Densities (bears/1000 km^2^) are based on total area of the DNA grid as listed in Table 1. The first model and estimates listed for each area is from the most supported SECR model. Other models and estimates are given to explore sensitivity to model assumptions. Closed model/telemetry estimates from original analyses^1^ are given for comparison.

| Model | |  | Expected N | | |  |  | Density | |  |  |
| --- | --- | --- | --- | --- | --- | --- | --- | --- | --- | --- | --- |
| Density | Detection | | $\hat{N}$ | SE | Conf. Int. | | CV | $\hat{D}$ | SE | Conf. Int. | |
| RSF*Risk | g_0_(TRI) σ(.) | | 119.2 | 9.1 | 103 | 138 | 7.7% | 6.11 | 0.47 | 5.28 | 7.08 |
| RSF | g_0_(TRI) σ(.) | | 118.3 | 9.3 | 101 | 138 | 7.9% | 6.07 | 0.48 | 5.18 | 7.08 |
| Constant | g_0_(TRI) σ(.) | | 134.2 | 10.1 | 115.8 | 155.5 | 7.5% | 6.88 | 0.52 | 5.94 | 7.97 |
| Constant | Closed N/Telemetry | | 136.1 | 21.91 | 110 | 204 | 16.1% | 6.98 | 1.12 | 5.64 | 10.46 |

## Yellowhead (BMA 3)

The Yellowhead BMA 3 bear management area was surveyed in 2004^2,11^. This survey employed both fixed and moved sites including a design where the first site placed was left for all 4 sessions in addition to a moved site in each cell as a means of testing the utility of moving sites^11^. Despite the high degree of survey effort only 24 females and 20 males were detected mainly upon the far western edge of the sampling grid (Table 8). We note that the total number of bears detected in Table 8 is higher than previous closed model analyses^2,11^ that only considered moved sites for estimates.

**Table 8: Summary statistics for the 2004 Alberta Yellowhead (Bear Management Area 3) which included both fixed and moved sites.**

|  | | **Session** | | | | | | | |  |
| --- | --- | --- | --- | --- | --- | --- | --- | --- | --- | --- |
| **Statistic** | | **1** | | **2** | | **3** | | **4** | **Total** | |
| ***Females*** | |  | |  | |  | |  |  | |
| Animals caught (n_j_) | | 7 | | 16 | | 16 | | 9 | 48 | |
| Newly caught (u_j_) | | 7 | | 11 | | 4 | | 2 | 24 | |
| Frequencies (f_j_) | | 10 | | 7 | | 4 | | 3 | 24 | |
| Total individuals caught (M_j_) | 7 | | 18 | | 22 | | 24 | | 24 | |
| Detections | | 13 | | 23 | | 18 | | 15 | 69 | |
| Detectors visited | | 12 | | 21 | | 14 | | 15 | 62 | |
| Detectors available | | 188 | | 365 | | 349 | | 360 | 1262 | |
| ***Males*** | |  | |  | |  | |  |  | |
| Animals caught (n_j_) | | 13 | | 10 | | 10 | | 8 | 41 | |
| Newly caught (u_j_) | | 13 | | 4 | | 1 | | 2 | 20 | |
| Frequencies (f_j_) | | 9 | | 2 | | 8 | | 1 | 20 | |
| Total individuals caught (M_j_) | 13 | | 17 | | 18 | | 20 | | 20 | |
| Detections | | 20 | | 21 | | 17 | | 9 | 67 | |
| Detectors visited | | 17 | | 18 | | 15 | | 7 | 57 | |
| Detectors available | | 188 | | 365 | | 349 | | 360 | 1262 | |

Initial analysis suggested that a buffer size of 40 kilometers was needed to minimize potential bias of density estimates. This buffer size was used for both male and female analyses.

### Females

Female detections mainly occurred on the western border of the sampling grid with few detections in the eastern section of the grid (Figure 5). The mean distance moved between detections of females was 6.5 km with an RSPV of 9.2 km with 69 detection events of 24 individual bears (Table 8).


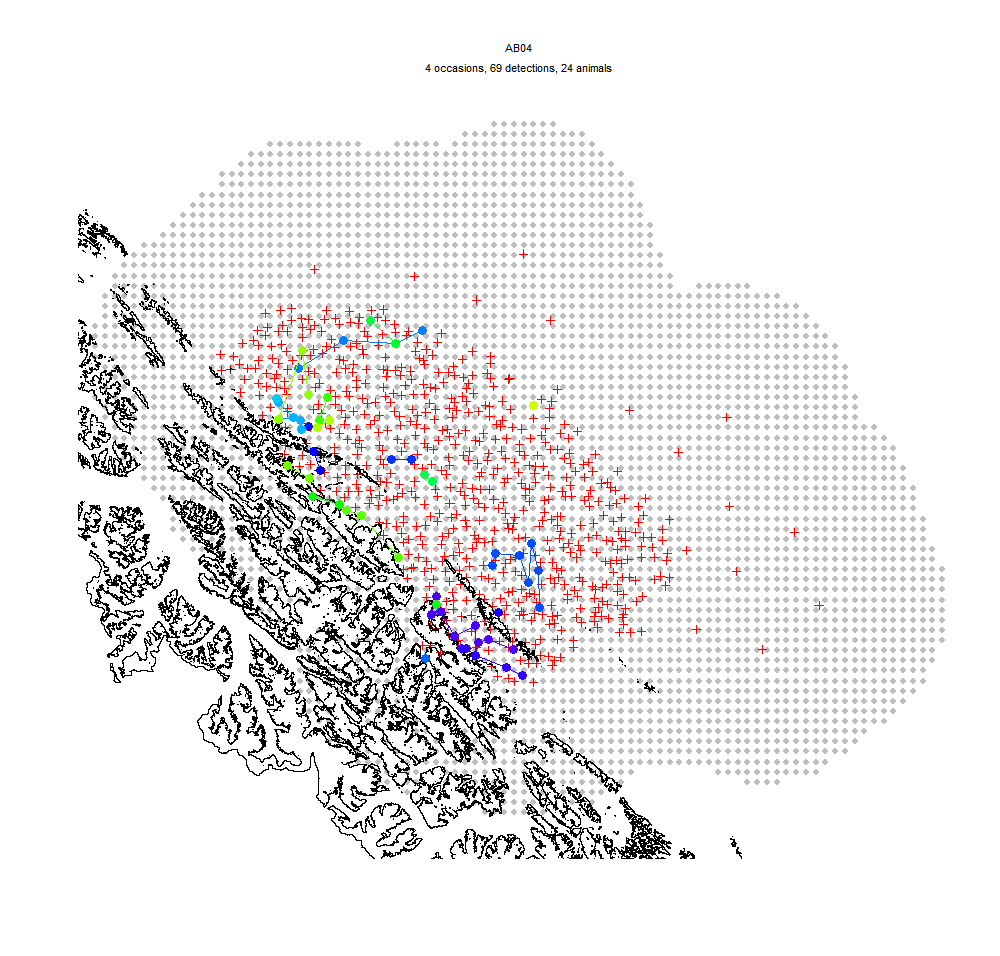


Figure 5: Detections and tracks of female grizzly bears in BMA 3 from repeated detections relative to habitat mask (grey dots), hair snag site locations (red + signs) and barren non-habitat (black polygons with no mask points inside them). The actual sequence of tracks from repeated detections does not necessarily reflect the true path of movements given that the time of within-session detection at hair snag sites is unknown. The cumulative locations of HS sites are displayed which include sites moved each session. Map was produced using the *secr* package ^6^ (v 2.9.2; <http://www.otago.ac.nz/density/SECRinR.html>) in program R^10^ (v 3.1.2; [www.r-project.org](http://www.r-project.org) ) plotting functions.

Initial model selection revealed an association between g_0_ and terrain ruggedness with constant σ values (Table 9, Model 4). The most supported density surface model suggested a positive association between RSF and density (Model 1).

Table 9: Abriged SECR model selection for females for the Yellowhead BMA 3 Inventory. AIC_c_ = sample size adjusted Akaike Information Criterion , ΔAIC_c_ = the difference in AIC_c_ between the model and the most supported model, AIC_c_ weight = w_i_, K, the number of model parameters and log-likelihood are given. Baseline constant models are shaded for reference with covariate models. The most supported baseline model (of models considered as listed in Table 2) is shown.

| No | Density | Detection | AIC_c_ | ∆AIC_c_ | w_i_ | K | LL |
| --- | --- | --- | --- | --- | --- | --- | --- |
| 1 | RSF | g_0_ (TRI) σ (.) | 631.17 | 0.00 | 0.75 | 5 | -308.9 |
| 2 | RSF+Risk | g_0_ (TRI) σ (.) | 633.81 | 2.64 | 0.20 | 6 | -308.4 |
| **3** | RSF+Risk+RSF*Risk | g_0_ (TRI) σ (.) | 636.70 | 5.54 | 0.05 | 7 | -307.9 |
| 4 | Constant | g_0_ (TRI) σ (.) | 647.70 | 16.53 | 0.00 | 4 | -318.8 |
| **5** | Risk | g_0_ (TRI) σ (.) | 650.51 | 19.35 | 0.00 | 5 | -318.6 |
| 6 | Constant | g_0_ (.) σ (.) | 664.76 | 33.59 | 0.00 | 3 | -328.8 |

A model with RSF as a predictor of density but with constant detection covariates (g_0_(.) σ(.)) was not supported (∆AIC_c_=8.9) further verifying terrain ruggedness as a detection covariate when habitat (RSF) was included in the density model.

A plot of estimated density and RSF score suggested increasing density with RSF score (Figure 6).

Figure 6: Estimated relationship between density and RSF scores from model 1 (Table 9) for females in BMA 3.

Estimates of expected population size were relatively similar between different model formulations with an increase in estimates if heterogeneity due to terrain ruggedness was included in the SECR model (Table 10). Sex-specific estimates of density were not produced from the original analysis of the BMA 3 data^2^.

Table 10: Estimates of expected population size and density for female grizzly bears in BMA 3. Densities (bears/1000 km^2^) are based on total area of the DNA grid as listed in Table 1. The first model and estimates listed for each area is from the most supported SECR model. Other models and estimates are given to explore sensitivity to model assumptions.

| Model |  | Expected population size | | | |  | Density |  |  |  |
| --- | --- | --- | --- | --- | --- | --- | --- | --- | --- | --- |
| Density | Detection | $\hat{N}$ | SE | Conf. Int. | | CV | $\hat{D}$ | SE | Conf. Int. | |
| RSF | g_0_ (TRI) σ (.) | 20.7 | 3.28 | 15.2 | 28.2 | 15.8% | 2.35 | 0.37 | 1.72 | 3.20 |
| RSF+Risk | g_0_ (TRI) σ (.) | 21.5 | 3.44 | 15.8 | 29.4 | 16.0% | 2.44 | 0.39 | 1.79 | 3.33 |
| Constant | g_0_ (TRI) σ (.) | 20.6 | 3.91 | 14.3 | 29.8 | 19.0% | 2.34 | 0.44 | 1.62 | 3.38 |
| Constant | g_0_ (.) σ (.) | 17.9 | 3.18 | 12.8 | 25.4 | 17.7% | 2.04 | 0.36 | 1.45 | 2.88 |

## Males

As with females, male detections mainly occurred on the western edge of the DNA sampling area with a few detections on the eastern section of the sampling grid. The mean distance moved between detections was 10.4 kilometers with an RPSV of 12.6 km from 67 detection events of 20 individual bears (Table 7).


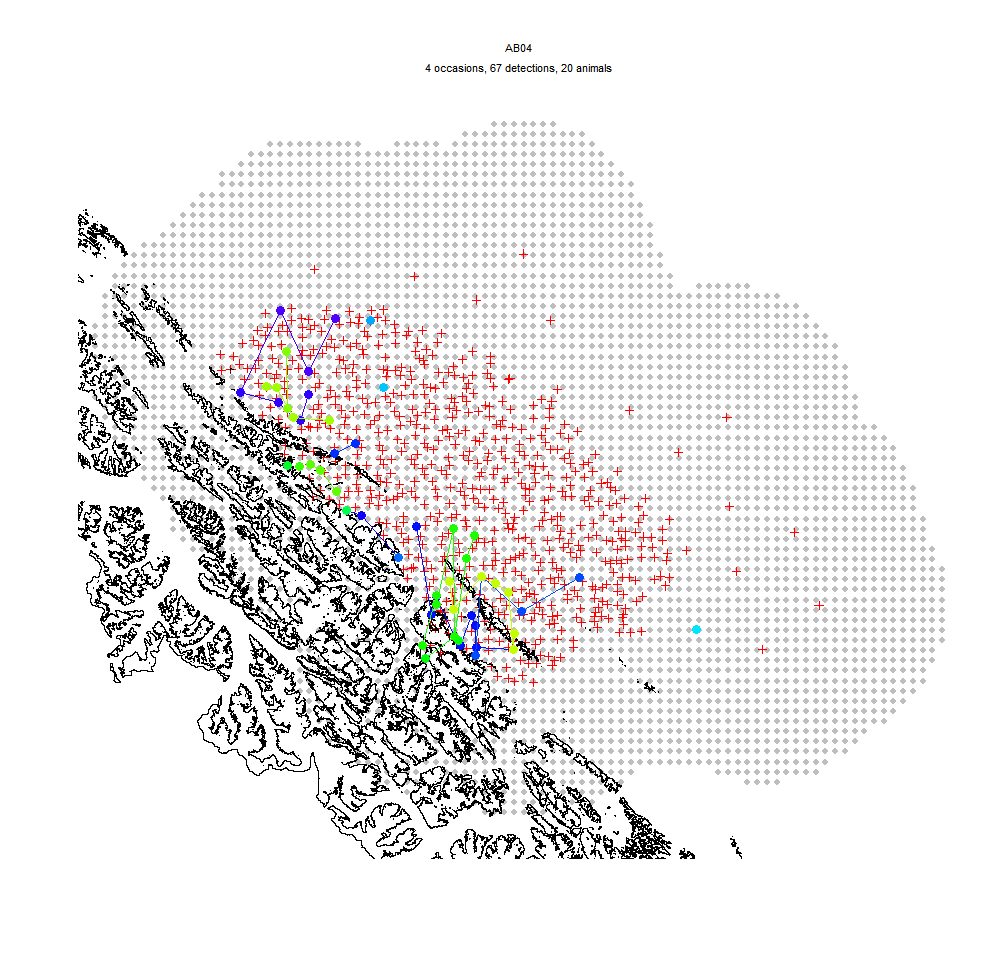


Figure 7: Detections and tracks of male grizzly bears in BMA 3 from repeated detections relative to habitat mask (grey dots), hair snag site locations (red + signs) and barren non-habitat (black polygons with no mask points inside them). The actual sequence of tracks from repeated detections does not necessarily reflect the true path of movements given that the time of within-session detection at hair snag sites is unknown. The cumulative locations of HS sites are displayed which include sites moved each session. Therefore, the actual spatial coverage of HS sites for one session will be overrepresented by this graphic. Map was produced using the *secr* package ^6^ (v 2.9.2; <http://www.otago.ac.nz/density/SECRinR.html>) in program R^10^ (v 3.1.2; [www.r-project.org](http://www.r-project.org) ) plotting functions.

Initial model selection revealed a positive association between scale of movement (σ) and terrain ruggedness as well as a temporal trend of decreasing scale of movement (Table 11, Model 5).

Table 11: Abriged SECR model selection for males for the Yellowhead BMA 3 Inventory. AIC_c_ = sample size adjusted Akaike Information Criterion, ΔAIC_c_ = the difference in AIC_c_ between the model and the most supported model, AIC_c_ weight = w_i_, K, the number of model parameters and log-likelihood are given. Baseline constant models are shaded for reference with covariate models. The most supported baseline model (of models considered as listed in Table 2) is shown.

| No | Density | Detection | AICc | ∆AICc | w_i_ | K | LL |
| --- | --- | --- | --- | --- | --- | --- | --- |
| 1 | RSF | g_0_ (.) σ (T+TRI) | 627.7 | 0.00 | 0.98 | 6 | -304.6 |
| 2 | RSF+Risk | g_0_ (.) σ (T+TRI) | 632.5 | 8.27 | 0.02 | 7 | -304.6 |
| 3 | RSF+Risk+RSF*Risk | g_0_ (.) σ (T+TRI) | 638.0 | 13.74 | 0.00 | 8 | -304.4 |
| 4 | Risk | g_0_ (.) σ (T+TRI) | 640.0 | 15.75 | 0.00 | 6 | -310.8 |
| 5 | Constant | g_0_ (.) σ (T+TRI) | 640.2 | 15.94 | 0.00 | 5 | -312.9 |
| 6 | Constant | g_0_ (.) σ (.) | 668.1 | 40.49 | 0.00 | 3 | -330.3 |

A model with RSF predicting density (Model 1) was most supported with reduced support for other density surface models. A model with RSF as a density predictor but with constant detection covariates had much lower support (∆AIC_c_=23.9) further supporting the inclusion of detection covariates (σ(T+TRI)). A plot of predicted density suggested an increase in density with RSF scores of greater than 0.2 (Figure 8).

Figure 8: Estimated relationship between density and RSF scores from males in BMA 3 from model 1 (Table 11).

Estimates of average population size and density increased when variation in σ was modelled with a slight increase in estimates when RSF score was used to model density variation (Table 12). Sex-specific estimates were not produced from the original analysis of the BMA 3 data^2^.

Table 12: Estimates of expected population size and density for male grizzly bears in BMA 3. Densities (bears/1000 km^2^) are based on total area of the DNA grid as listed in Table 1. The first model and estimates listed for each area is from the most supported SECR model. Other models and estimates are given to explore sensitivity to model assumptions.

| Model |  | Expected population size | | | |  | Density | |  |  |
| --- | --- | --- | --- | --- | --- | --- | --- | --- | --- | --- |
| Density | Detection | $\hat{N}$ | SE | Conf. Int. | | CV | $\hat{D}$ | SE | Conf. Int. | |
| RSF | g_0_(.) σ (T+TRI) | 14.5 | 2.40 | 10.52 | 20.02 | 16.5% | 1.64 | 0.27 | 1.19 | 2.27 |
| RSF+Risk | g_0_(.) σ (T+TRI) | 14.4 | 2.50 | 10.27 | 20.16 | 17.4% | 1.63 | 0.28 | 1.16 | 2.29 |
| Constant | g_0_(.) σ (T+TRI) | 12.7 | 2.42 | 8.77 | 18.39 | 19.1% | 1.44 | 0.27 | 0.99 | 2.09 |
| Constant | g0(.) σ (.) | 11.9 | 2.23 | 8.33 | 17.2 | 18.7% | 1.35 | 0.25 | 0.94 | 1.95 |

## Clearwater (BMA 4)

The Clearwater (BMA 4) area was sampled in 2005^3^ (Table 13). Initial analyses were conducted with sexes pooled to assess appropriate buffer distances to negate bias in density estimates due to movements from the grid area. A buffer size of 15 kilometers was estimated using the esa.plot and suggest.buffer functions in program SECR. As with other analyses 3.5 kilometer mask centroid spacing was used.

**Table 13: Summary statistics for the 2005 Alberta Clearwater (Bear Management BMA 4)**

|  | | **Session** | | | | | | | |  |
| --- | --- | --- | --- | --- | --- | --- | --- | --- | --- | --- |
| **Statistic** | | **1** | | **2** | | **3** | | **4** | **Total** | |
| ***Females*** | |  | |  | |  | |  |  | |
| Animals caught (n_j_) | | 10 | | 15 | | 13 | | 19 | 57 | |
| Newly caught (u_j_) | | 10 | | 9 | | 4 | | 2 | 25 | |
| Frequencies (f_j_) | | 7 | | 8 | | 6 | | 4 | 25 | |
| Total individuals caught (M_j_) | 10 | | 19 | | 23 | | 25 | | 25 | |
| Detections | | 11 | | 18 | | 18 | | 22 | 69 | |
| Detectors visited | | 9 | | 15 | | 16 | | 17 | 57 | |
| Detectors available | | 187 | | 183 | | 181 | | 182 | 733 | |
| ***Males*** | |  | |  | |  | |  |  | |
| Animals caught (n_j_) | | 7 | | 15 | | 12 | | 8 | 42 | |
| Newly caught (u_j_) | | 7 | | 9 | | 1 | | 0 | 17 | |
| Frequencies (f_j_) | | 5 | | 2 | | 7 | | 3 | 17 | |
| Total individuals caught (M_j_) | 7 | | 16 | | 17 | | 17 | | 17 | |
| Detections | | 16 | | 26 | | 19 | | 10 | 71 | |
| Detectors visited | | 14 | | 24 | | 19 | | 10 | 67 | |
| Detectors available | | 187 | | 183 | | 181 | | 182 | 733 | |

### Females

The distribution of females was mainly centered in mountainous areas with few bears detected on the eastern part of the sampling grid (Figure 9). Overall, 25 females were detected with 69 unique detection events. From this the estimated movement between detections was 8.2 km with an RPSV of 7.9 km.


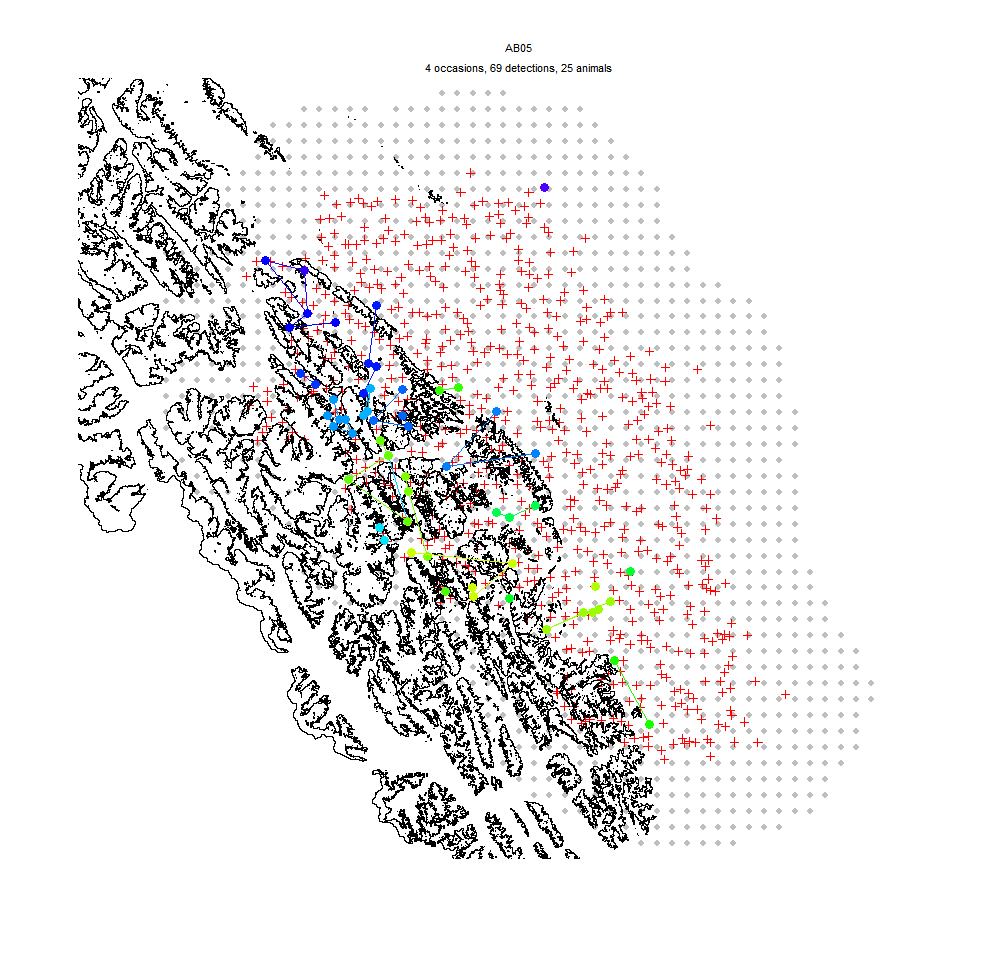


Figure 9: Detections and tracks of female grizzly bears in BMA 4 from repeated detections relative to habitat mask (grey dots), hair snag site locations (red + signs) and barren non-habitat (black polygons with no mask points inside them). The actual sequence of tracks from repeated detections does not necessarily reflect the true path of movements given that the time of within-session detection at hair snag sites is unknown. The cumulative locations of HS sites are displayed which include sites moved each session. Therefore, the actual spatial coverage of HS sites for one session will be overrepresented by this graphic. Map was produced using the *secr* package ^6^ (v 2.9.2; <http://www.otago.ac.nz/density/SECRinR.html>) in program R^10^ (v 3.1.2; [www.r-project.org](http://www.r-project.org) ) plotting functions.

Model selection results suggested a temporal increasing trend in movement of grizzly bears with detection probabilities at the home range center being positivity related to terrain ruggedness (Model 4, Table 14). Of density surface models, a model with RSF was most related to density of grizzly bears on the sampling grid (Model 1).

Table 14: Abridged model selection for female grizzly bear SECR analysis for BMA 4 (2005). AIC_c_ = sample size adjusted Akaike Information Criterion, ΔAIC_c_ = the difference in AIC_c_ between the model and the most supported model, AIC_c_ weight = w_i_, K, the number of model parameters and log-likelihood are given. Baseline constant models are shaded for reference with covariate models. The most supported baseline model (of models considered as listed in Table 2) is shown.

| No | Density | Detection | AICc | ΔAIC_c_ | w_i_ | K | LL |
| --- | --- | --- | --- | --- | --- | --- | --- |
| 1 | RSF | g_0_ (TRI) σ (T) | 564.1 | 0.00 | 0.86 | 6 | -273.7 |
| 2 | RSF +Risk | g_0_ (TRI) σ (T) | 568.0 | 3.89 | 0.12 | 7 | -273.7 |
| 3 | RSF +Risk +RSF*Risk | g_0_ (TRI) σ (T) | 572.2 | 8.07 | 0.02 | 8 | -273.6 |
| 4 | Constant | g_0_ (TRI) σ (T) | 576.0 | 11.87 | 0.00 | 5 | -281.4 |
| 5 | Risk | g_0_ (TRI) σ (T) | 579.5 | 15.37 | 0.00 | 6 | -281.4 |
| 6 | Constant | Constant | 589.4 | 25.30 | 0.00 | 3 | -291.1 |

A model with RSF as a predictor of density and constant detection parameters (g0(.) σ(.)) was less supported (∆AIC_c_=3.99) further suggesting support for the detection covariates.

A plot of prediction by RSF score from Model 1 suggested lower densities at mask centroids with RSF scores of less than 0.2 and large increase at higher RSF scores (Figure 10).

**
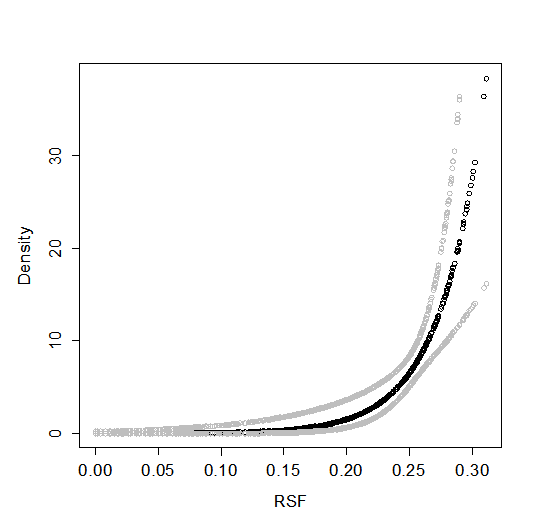
**

Figure 10: Relationship between Density and RSF score for female bears in BMA 4(Model 1, Table 14)

Estimates of population size and density exhibited lower densities for RSF surface models in comparison to models that assumed constant density (Table 15). Estimates of sex-specific density were not produced in the original analysis of the BMA 4 data set^3^.

Table 15: Estimates of expected population size and density for female grizzly bears in BMA 4. Densities (bears/1000 km^2^) are based on total area of the DNA grid as listed in Table 1. The first model and estimates listed for each area is from the most supported SECR model. Other models and estimates are given to explore sensitivity to model assumptions.

| Model |  | Expected population size | | | |  | Density | |  |  |
| --- | --- | --- | --- | --- | --- | --- | --- | --- | --- | --- |
| Density | Detection | $\hat{N}$ | SE | Conf. Int. | | CV | $\hat{D}$ | SE | Conf. Int. | |
| RSF | g_0_(TRI) σ (T) | 21.6 | 2.6 | 17.0 | 27.4 | 12.2% | 2.40 | 0.29 | 1.89 | 3.04 |
| Constant | g_0_(TRI) σ (T) | 31.4 | 6.6 | 20.9 | 47.2 | 21.1% | 3.48 | 0.73 | 2.32 | 5.24 |
| RSF | g_0_(.) σ (.) | 21.3 | 2.5 | 16.9 | 26.8 | 11.9% | 2.36 | 0.28 | 1.87 | 2.97 |

### Males

Males were also distributed closer to the mountainous areas with some paths leading to eastern sections of the grid. Overall 17 males were detected with 71 detection events throughout the survey (Figure 11). The mean distance moved between detections for male was 14.7 km with an RSPV of 16.5 km. Some larger scale movements were apparent in the central part of the sampling grid.


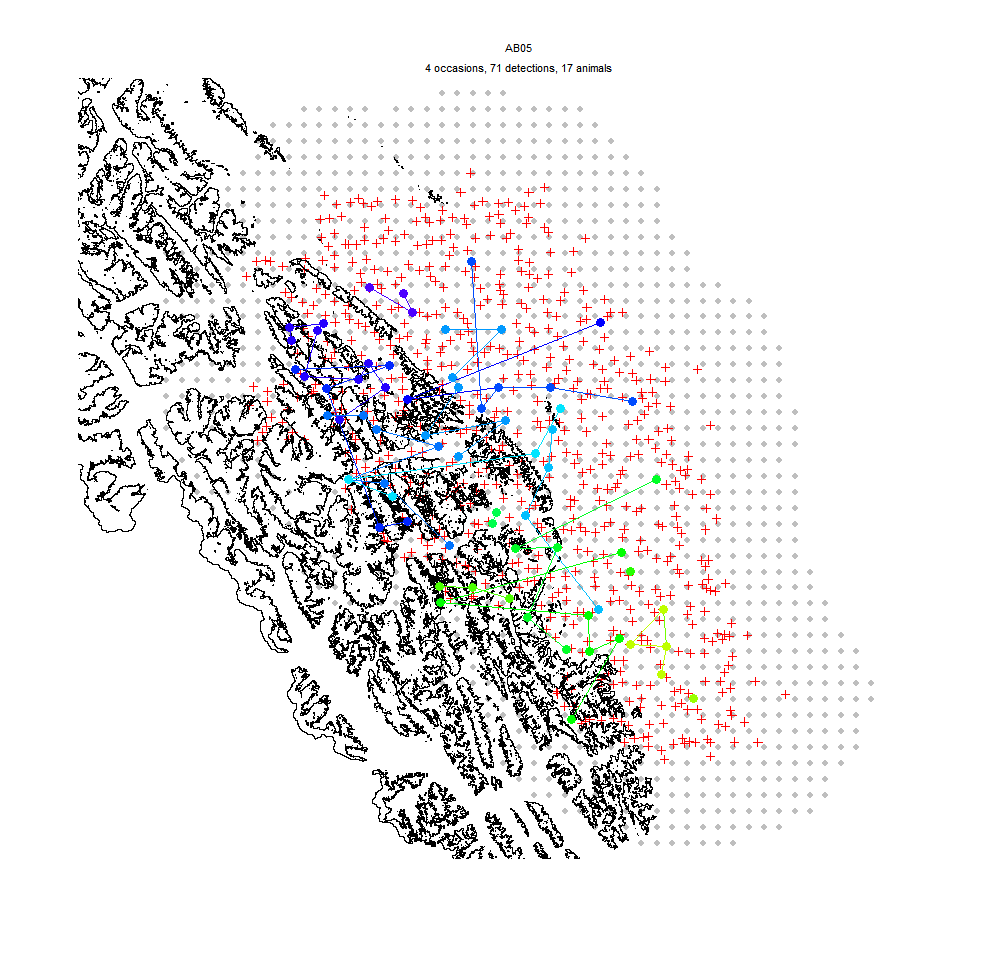


Figure 11: Detections and tracks of male grizzly bears in BMA 4 from repeated detections relative to habitat mask (grey dots), hair snag site locations (red + signs) and barren non-habitat (black polygons with no mask points inside them). The actual sequence of tracks from repeated detections does not necessarily reflect the true path of movements given that the time of within-session detection at hair snag sites is unknown. The cumulative locations of HS sites are displayed which include sites moved each session. Therefore, the actual spatial coverage of HS sites for one session will be overrepresented by this graphic. Map was produced using the *secr* package ^6^ (v 2.9.2; <http://www.otago.ac.nz/density/SECRinR.html>) in program R^10^ (v 3.1.2; [www.r-project.org](http://www.r-project.org) ) plotting functions.

Model selection results suggested that detection of male bears was positively related to terrain ruggedness with scale of movement being relatively constant (Table 16 Model 2). Of density surface models, a model that associated density with RSF score on the logit scale was most supported (Table 16, Model 1).

Table 16: Abridged model selection for male grizzly bears for the BMA 4 (2005) inventory. AIC_c_ = sample size adjusted Akaike Information Criterion, ΔAIC_c_ = the difference in AIC_c_ between the model and the most supported model, AIC_c_ weight = w_i_, K, the number of model parameters and log-likelihood are given. Baseline constant models are shaded for reference with covariate models. The most supported baseline model (of models considered as listed in Table 2) is shown.

| No | Density | Detection | AIC_c_ | ΔAIC_c_ | w_i_ | K | LL |
| --- | --- | --- | --- | --- | --- | --- | --- |
| 1 | RSF | g_0_ (TRI) σ (.) | 693.16 | 0.00 | 0.70 | 5 | -338.9 |
| 2 | constant | g_0_ (TRI) σ (.) | 696.89 | 3.73 | 0.11 | 4 | -342.8 |
| 3 | RSF +Risk | g_0_ (TRI) σ (.) | 697.84 | 4.69 | 0.11 | 6 | -338.7 |
| 4 | Risk | g_0_ (TRI) σ (.) | 700.46 | 7.31 | 0.07 | 5 | -342.5 |
| 5 | constant | g_0_ (.) σ (.) | 702.33 | 9.18 | 0.02 | 3 | -347.2 |
| 6 | RSF +Risk +RSF*Risk | g_0_ (TRI) σ (.) | 703.85 | 10.7 | 0.0 | 7 | -341.6 |

A model with RSF as a predictor of density but with constant detection parameters (g_0_(.) σ(.)) was less supported (∆AIC=1.05) suggesting support for TRI as a covariate for g_0_ when habitat was included in the density surface model.

Model 1 (Table 16) predicted male density increasing with RSF score especially with RSF values above 0.15 (Figure 12).

Figure 12: The predicted relationship between density and RSF score for male bears in BMA 4 from Model 1 (Table 16).

Estimates of population size and density were slightly higher for models with a density surface compared to constant density models (Table 17). The estimate of expected population size was lower than the total number of males detected on the grid which was presumably due to a larger number of bears inhabiting the western edge of the sampling grid. Estimates of sex-specific density were not produced in the original analysis of the BMA 4 data set^3^.

Table 17: Estimates of expected population size and density for male grizzly bears for BMA 4 Densities (bears/1000 km^2^) are based on total area of the DNA grid as listed in Table 1. The first model and estimates listed for each area is from the most supported SECR model. Other models and estimates are given to explore sensitivity to model assumptions.

| Model |  | | Expected population size | | | |  | Density | | | |
| --- | --- | --- | --- | --- | --- | --- | --- | --- | --- | --- | --- |
| Density | | Detection | $\hat{N}$ | SE | Conf. Int. | | CV | $\hat{D}$ | SE | Conf. Int. | |
| RSF | | g_0_(TRI) σ (.) | 13.5 | 1.4 | 11.0 | 16.4 | 10.1% | 1.50 | 0.16 | 1.22 | 1.82 |
| constant | | g_0_(TRI) σ (.) | 11.9 | 1.7 | 9.0 | 15.8 | 14.4% | 1.32 | 0.19 | 1.00 | 1.75 |
| RSF | | g_0_(.) σ (.) | 13.7 | 1.3 | 11.4 | 16.6 | 9.6% | 1.52 | 0.14 | 1.26 | 1.84 |

## Livingston (BMA 5)

The Livingston (BMA 5) bear management unit (Table 18) was sampled in 2006^4^. Initial analyses were conducted with sexes pooled to assess appropriate buffer distances to negate bias in density estimates due to movements from the grid area. A buffer size of 35 kilometers was estimated using the esa.plot and suggest.buffer functions in program secr. This larger buffer size was due to long-range movements of males along the sampling grid. Due to issues with missing landcover data for some DNA sites, site covariates were not run for the 2006 data set analysis.

**Table 18: Summary statistics for the 2006 Alberta Livingston (Bear Management Area 5)**

|  | | **Session** | | | | | | | |  |
| --- | --- | --- | --- | --- | --- | --- | --- | --- | --- | --- |
| **Statistic** | | **1** | | **2** | | **3** | | **4** | **Total** | |
| ***Females*** | |  | |  | |  | |  |  | |
| Animals caught (n_j_) | | 14 | | 17 | | 22 | | 21 | 74 | |
| Newly caught (u_j_) | | 14 | | 13 | | 12 | | 6 | 45 | |
| Frequencies (f_j_) | | 24 | | 14 | | 6 | | 1 | 45 | |
| Total individuals caught (M_j_) | 14 | | 27 | | 39 | | 45 | | 45 | |
| Detections | | 17 | | 21 | | 27 | | 26 | 91 | |
| Detectors visited | | 15 | | 18 | | 21 | | 23 | 77 | |
| Detectors available | | 165 | | 155 | | 160 | | 158 | 638 | |
| ***Males*** | |  | |  | |  | |  |  | |
| Animals caught (n_j_) | | 16 | | 13 | | 18 | | 13 | 60 | |
| Newly caught (u_j_) | | 16 | | 8 | | 10 | | 6 | 40 | |
| Frequencies (f_j_) | | 25 | | 11 | | 3 | | 1 | 40 | |
| Total individuals caught (M_j_) | 16 | | 24 | | 34 | | 40 | | 40 | |
| Detections | | 19 | | 17 | | 26 | | 15 | 77 | |
| Detectors visited | | 15 | | 17 | | 16 | | 12 | 60 | |
| Detectors available | | 165 | | 155 | | 160 | | 158 | 638 | |

### Females

Female grizzly bears were mainly distributed in mountainous areas with moderate distances travelled between detections. Overall, 45 individuals were identified with 91 detection events (Figure 13). The mean distance moved between detections was 6.5 km with an RSPV of 6.5 km from the 91 detection events.


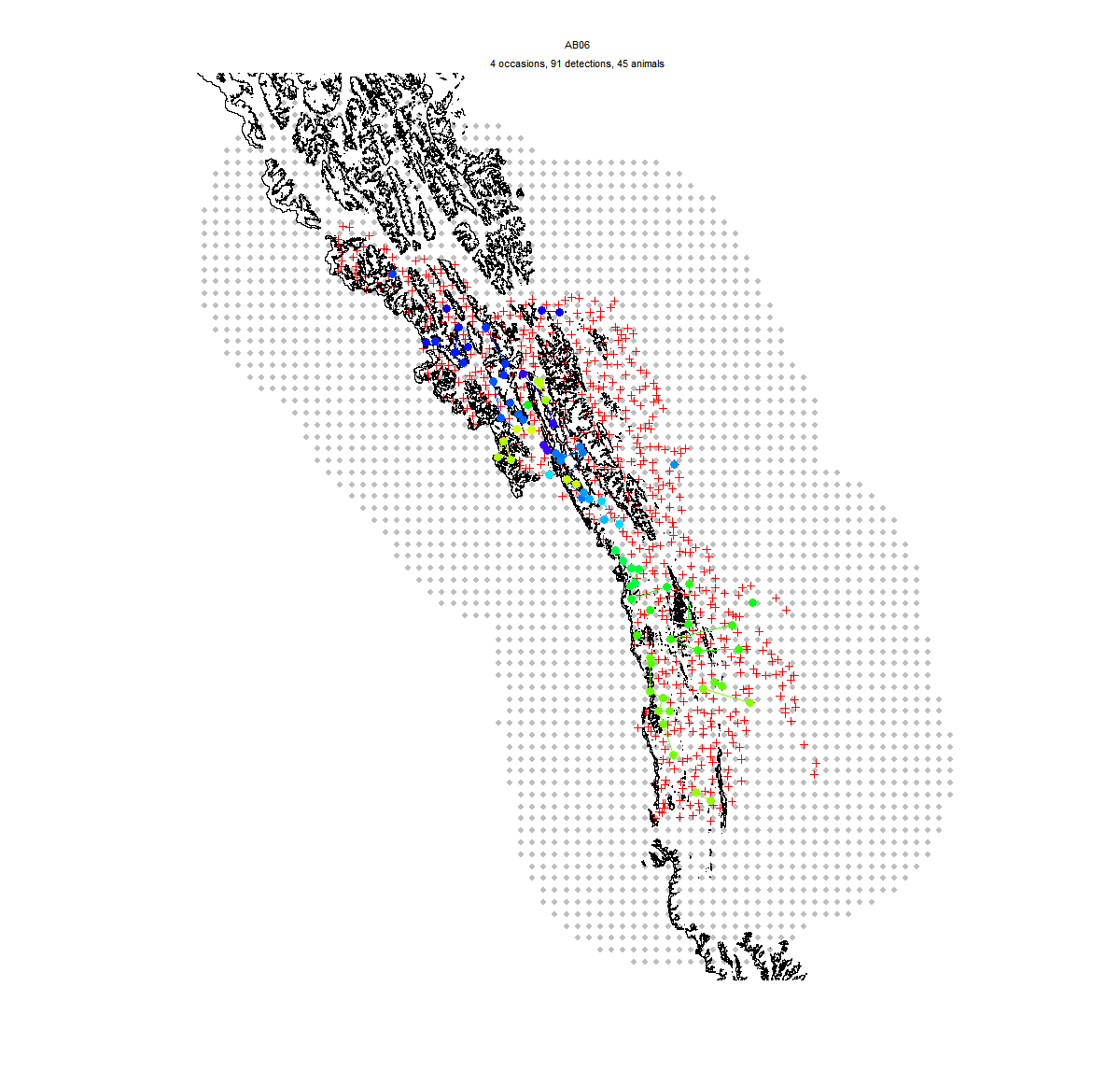


Figure 13: Detections and tracks of female grizzly bears in BMA 5 from repeated detections relative to habitat mask (grey dots), hair snag site locations (red + signs) and barren non-habitat (black polygons with no mask points inside them). The actual sequence of tracks from repeated detections does not necessarily reflect the true path of movements given that the time of within-session detection at hair snag sites is unknown. The cumulative locations of HS sites are displayed which include sites moved each session. Therefore, the actual spatial coverage of HS sites for one session will be overrepresented by this graphic. Map was produced using the *secr* package ^6^ (v 2.9.2; <http://www.otago.ac.nz/density/SECRinR.html>) in program R^10^ (v 3.1.2; [www.r-project.org](http://www.r-project.org) ) plotting functions.

A model with a linear increasing trend in increase detection at home range center and constant scale of movement was most supported (Table 19, Model 4). Of density surface models, a model with density associated with risk was most supported (Model 1).

Table 19: Abridged model selection results for females for BMA 5 Inventory. AIC_c_ = sample size adjusted Akaike Information Criterion, ΔAIC_c_ = the difference in AIC_c_ between the model and the most supported model, AIC_c_ weight = w_i_, K, the number of model parameters and log-likelihood are given. Baseline constant models are shaded for reference with covariate models. The most supported baseline model (of models considered as listed in Table 2) is shown.

| No | Density | Detection | AICc | ΔAIC_c_ | w_i_ | K | LL |
| --- | --- | --- | --- | --- | --- | --- | --- |
| 1 | Risk | g_0_ (T) σ (.) | 728.11 | 0.00 | 0.65 | 5 | -358.3 |
| 2 | RSF +Risk | g_0_ (T) σ (.) | 730.59 | 2.48 | 0.19 | 6 | -358.2 |
| 3 | RSF +Risk +RSF*Risk | g_0_ (T) σ (.) | 731.94 | 3.83 | 0.10 | 7 | -357.5 |
| 4 | constant | g_0_ (T) σ (.) | 733.89 | 5.77 | 0.04 | 4 | -362.4 |
| 5 | constant | g_0_ (.) σ (.) | 734.67 | 6.56 | 0.02 | 3 | -364.0 |
| 6 | RSF | g_0_ (T) σ (.) | 736.25 | 8.14 | 0.01 | 5 | -362.4 |

A model with Risk as a predictor of density and constant detection parameters (g_0_(.) σ(.)) was less supported (∆AIC_c_=0.2) further supporting a linear trend in g_0_.

Plots of density predictions defined a gradual reduction in density with increasing risk (Figure 14).

Figure 14: Predicted density as a function of risk score from Model 1 (Table 19) for females in BMA 5 inventory

Estimates of expected population size were similar for the grid area (Table 20).

Table 20: Estimates of expected population size and density for females on the BMA 5 DNA inventory area. Densities (bears/1000 km^2^) are based on total area of the DNA grid as listed in Table 1 The first model and estimates listed for each area is from the most supported SECR model. Other models and estimates are given to explore sensitivity to model assumptions.

| SECR model | | Expected Population size | | | |  | Density | | |  |
| --- | --- | --- | --- | --- | --- | --- | --- | --- | --- | --- |
| Density | Detection | $\hat{N}$ | SE | Conf. Int. | | CV | $\hat{D}$ | SE | Conf. Int. | |
| RISK | g_0_ (T) σ (.) | 46.7 | 6.7 | 35.3 | 61.8 | 14.3% | 5.74 | 0.82 | 4.34 | 7.59 |
| RSF RISK | g_0_ (T) σ (.) | 47.2 | 6.8 | 35.6 | 62.5 | 14.4% | 5.80 | 0.84 | 4.37 | 7.68 |
| constant | g_0_ (T) σ (.) | 44.1 | 6.6 | 33.0 | 59.0 | 15.0% | 5.42 | 0.81 | 4.05 | 7.24 |

### Males

Males were also mainly distributed in the western mountainous area of the sampling grid (Figure 15). One individual moved across the northern border of the sampling grid. This longer movement inflated σ values which resulted in the larger buffer distance required for this analysis.


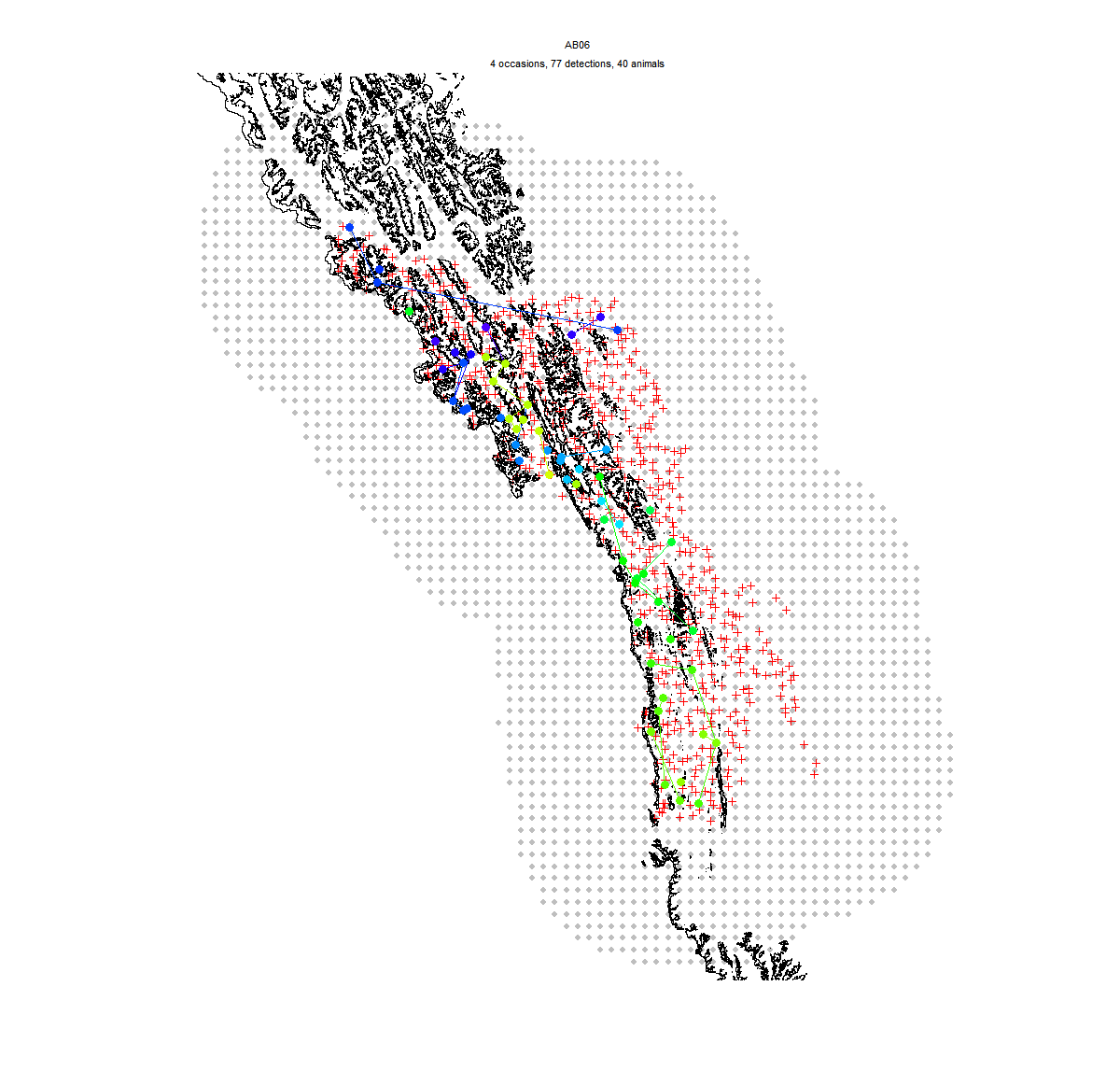


Figure 15: Detections and tracks of male grizzly bears in BMA 5 from repeated detections relative to habitat mask (grey dots), hair snag site locations (red + signs) and barren non-habitat (black polygons with no mask points inside them). The actual sequence of tracks from repeated detections does not necessarily reflect the true path of movements given that the time of within-session detection at hair snag sites is unknown. The cumulative locations of HS sites are displayed which include sites moved each session. Therefore, the actual spatial coverage of HS sites for one session will be overrepresented by this graphic. Map was produced using the *secr* package ^6^ (v 2.9.2; <http://www.otago.ac.nz/density/SECRinR.html>) in program R^10^ (v 3.1.2; [www.r-project.org](http://www.r-project.org) ) plotting functions.

As with females, site covariates were not used for the analysis. Of base models considered, a model with constant detection probabilities at home range center and scale varying heterogeneously as a mixture model was most supported (Table 21, Model 7). This model estimated varying levels of σ which was presumably due to the large movement distance of some males on the survey grid (Figure 20). Of density surface models, a model with density varying with risk was most supported (Table 21, Model 1).

Table 21: Abridged Male model selection for 2006 BMA 5 Inventory project. Baseline constant models are shaded for reference with covariate models. AIC_c_ = sample size adjusted Akaike Information Criterion , ΔAIC_c_ = the difference in AIC_c_ between the model and the most supported model, AIC_c_ weight = w_i_, K, the number of model parameters and log-likelihood are given. Baseline constant models are shaded for reference with covariate models. The most supported baseline model (of models considered as listed in Table 2) is shown.

| No | Density | Detection | AICc | ΔAIC_c_ | w_i_ | K | LL |
| --- | --- | --- | --- | --- | --- | --- | --- |
| 1 | Risk | g_0_(.) σ (h_2_) | 704.71 | 0.00 | 0.44 | 6 | -345.1 |
| 2 | RSF +Risk | g_0_(.) σ (h_2_) | 704.87 | 0.16 | 0.41 | 7 | -343.7 |
| 3 | RSF+Risk +RSF*Risk | g_0_(.) σ (h_2_) | 707.00 | 2.29 | 0.14 | 8 | -343.2 |
| 4 | constant | g_0_(.) σ (h_2_) | 713.65 | 8.93 | 0.01 | 5 | -350.9 |
| 5 | RSF | g_0_(.) σ (h_2_) | 714.78 | 10.07 | 0.00 | 6 | -350.1 |
| 6 | constant | g_0_(.) σ (.) | 730.25 | 25.54 | 0.00 | 3 | -361.8 |

A model with Risk as a density covariate but with constant detection parameters (g0(.) σ(.)) was less supported than Model 1 with the σ mixture model (∆AICc=14.4) further supporting the use of the σ mixture model.

A plot of density predictions from model 1 predict density decreases as risk increases (Figure 16).

Figure 16: Predicted density as a function of risk level for male bears in BMA 5 (Model 1, Table 20)

Estimates were relatively similar between various model formulations. The mixture model used to model variation in σ increased estimates slightly (Table 22).

Table 22: Expected population size and density for male bears on the BMA 5 sampling grid. Densities (bears/1000 km^2^) are based on total area of the DNA grid as listed in Table 1. The first model and estimates listed for each area is from the most supported SECR model. Other models and estimates are given to explore sensitivity to model assumptions.

| SECR Model | | Expected Population size | | | |  | Density | | | |
| --- | --- | --- | --- | --- | --- | --- | --- | --- | --- | --- |
| Density | Detection | $\hat{N}$ | SE | Conf. Int. | | CV | $\hat{D}$ | SE | Conf. Int. | |
| RISK | g_0_(.) σ (h_2_) | 34.5 | 5.6 | 25.1 | 47.3 | 16.3% | 4.24 | 0.69 | 3.09 | 5.82 |
| RSF RISK | g_0_(.) σ (h_2_) | 29.6 | 6.2 | 19.6 | 44.5 | 21.1% | 3.63 | 0.77 | 2.41 | 5.47 |
| Constant | g_0_(.) σ (h_2_) | 31.6 | 5.6 | 22.5 | 44.5 | 17.6% | 3.88 | 0.69 | 2.77 | 5.47 |
| RISK | constant | 28.9 | 4.7 | 21.1 | 39.5 | 16.2% | 3.55 | 0.58 | 2.59 | 4.86 |

##

## Castle BMA 6 (2007)

The Castle BMA 6 grizzly bear inventory project was conducted in 2007^5^. Areas in British Columbia and Alberta were sampled during the 2007 project. For this analysis, we only considered the Alberta portion of the data set (Table 23). For preliminary analyses, a model with sex defined as groups was run to estimate a buffer area of 8.2 kilometers needed to minimize bias in density estimates. As with other analyses, a buffer centroid spacing of 3.5 kilometers was used for the mask. As with other analyses 3.5 kilometer mask centroid spacing was used. A sensitivity analysis was conducted to determine if density estimates changed in mask spacing was reduced with minimal difference if mask spacing was increased.

**Table 23: Summary statistics for the 2007 Alberta Castle (Bear Management BMA 6)**

|  | | **Session** | | | | | | | |  |
| --- | --- | --- | --- | --- | --- | --- | --- | --- | --- | --- |
| **Statistic** | | **1** | | **2** | | **3** | | **4** | **Total** | |
| ***Females*** | |  | |  | |  | |  |  | |
| Animals caught (n_j_) | | 2 | | 5 | | 0 | | 8 | 15 | |
| Newly caught (u_j_) | | 2 | | 5 | | 0 | | 6 | 13 | |
| Frequencies (f_j_) | | 11 | | 2 | | 0 | | 0 | 13 | |
| Total individuals caught (M_j_) | 2 | | 7 | | 7 | | 13 | | 13 | |
| Detections | | 2 | | 8 | | 0 | | 9 | 19 | |
| Detectors visited | | 2 | | 8 | | 0 | | 7 | 17 | |
| Detectors available | | 57 | | 58 | | 56 | | 57 | 228 | |
| ***Males*** | |  | |  | |  | |  |  | |
| Animals caught (n_j_) | | 4 | | 10 | | 4 | | 6 | 24 | |
| Newly caught (u_j_) | | 4 | | 9 | | 3 | | 3 | 19 | |
| Frequencies (f_j_) | | 15 | | 3 | | 1 | | 0 | 19 | |
| Total individuals caught (M_j_) | 4 | | 13 | | 16 | | 19 | | 19 | |
| Detections | | 4 | | 11 | | 4 | | 7 | 26 | |
| Detectors visited | | 4 | | 8 | | 4 | | 5 | 21 | |
| Detectors available | | 57 | | 58 | | 56 | | 57 | 228 | |

### Females

Compared to other projects, there were relatively few redetections of female bears with minimal movements recorded between redetections (Figure 17). All but one female was detected on the western fringe of the sampling grid. Overall 13 bears were detected with only 19 detection events. The mean distance moved between detections was 4.4 km with and RSPV of 4.2 km for the 19 detection events.


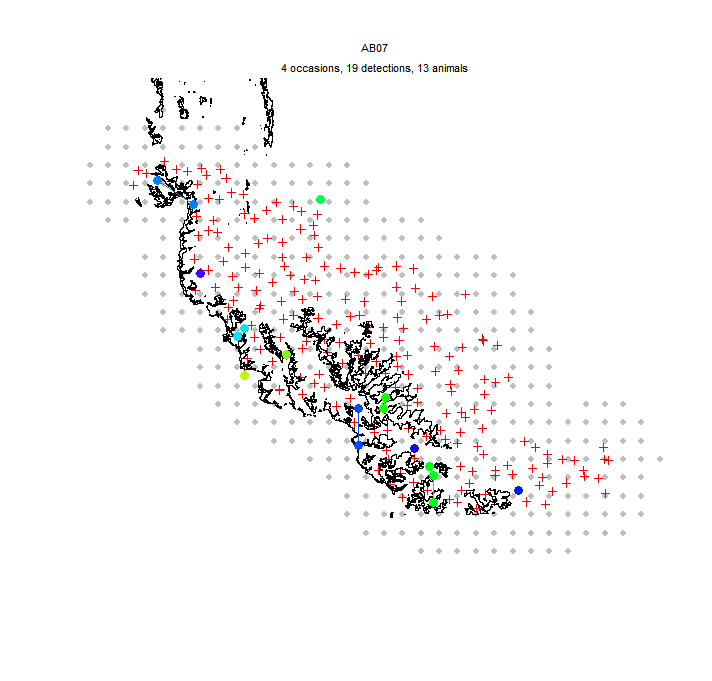


Figure 17: : Detections and tracks of female grizzly bears in BMA 6 from repeated detections relative to habitat mask (grey dots), hair snag site locations (red + signs) and barren non-habitat (black polygons with no mask points inside them). The actual sequence of tracks from repeated detections does not necessarily reflect the true path of movements given that the time of within-session detection at hair snag sites is unknown. The cumulative locations of HS sites are displayed which include sites moved each session. Therefore, the actual spatial coverage of HS sites for one session will be overrepresented by this graphic. Map was produced using the *secr* package ^6^ (v 2.9.2; <http://www.otago.ac.nz/density/SECRinR.html>) in program R^10^ (v 3.1.2; [www.r-project.org](http://www.r-project.org) ) plotting functions.

Model selection initially focused on the most parsimonious detection and scale covariates. Of these, a model with detection at home range center varying positively with terrain ruggedness index (at the site scale) and constant scale of movement was most supported (Table 24, model 2). Using this model as a base, density surface models were considered. Of these, a model with risk was most supported (Model 1).

Table 24: Abridged model selection for female grizzly bears for the BMA 6 inventory. AIC_c_ = sample size adjusted Akaike Information Criterion, ΔAIC_c_ = the difference in AIC_c_ between the model and the most supported model, AIC_c_ weight = w_i_, K, the number of model parameters and log-likelihood are given. Baseline constant models are shaded for reference with covariate models. The most supported baseline model (of models considered as listed in Table 2) is shown.

| No | Density | Detection | AIC_c_ | ΔAIC_c_ | w_i_ | K | LL |
| --- | --- | --- | --- | --- | --- | --- | --- |
| 1 | Risk | g_0_(TRI) σ (.) | 135.9 | 0.00 | 0.81 | 5 | -58.7 |
| 2 | constant | g_0_(TRI) σ (.) | 139.6 | 3.66 | 0.13 | 4 | -63.3 |
| 3 | RSF +Risk | g_0_(TRI) σ (.) | 142.1 | 6.15 | 0.04 | 6 | -58.0 |
| 4 | RSF | g_0_(TRI) σ (.) | 142.6 | 6.72 | 0.03 | 5 | -62.0 |
| 5 | RSF +Risk +RSF*Risk | g_0_(TRI) σ (.) | 152.4 | 16.51 | 0.00 | 7 | -58.0 |
| 6 | constant | constant | 155.7 | 19.73 | 0.00 | 3 | -73.5 |

A model with RISK as a density covariate but with constant detection parameters (g0(.) σ(.)) was less supported (∆AICc=3.59) further supporting TRI as a covariate for g_0_ (Model 1).

A plot of density versus risk suggests higher bear densities in areas of risk of less than 0.4 (Figure 18).

Figure 18: The relationship between risk and bear density from Model 1 (Table 15)

Estimates varied between base models as well as density surface models with a tendency towards higher estimates if even density was assumed (Table 25). Estimates were also imprecise therefore making it difficult to determine if apparent differences were simply due to lack of overall estimate precision. Estimates were lower than historic closed N telemetry estimates, however, low estimate precision as well as low numbers of collared bears needed to scale closed estimates makes these estimates less reliable. Estimates were also run at 2 km mask spacing to check if the relatively large 3.5 km spacing effected estimates. Estimates from the D(.) g_0_(TRI) σ (.) for the DNA sampling grid was 23.1 (SE=6.8, CI=13.1-40.7). These estimates were slightly different than the 3.5 km grid spacing suggesting that grid cell spacing had a marginal effect on estimates.

Table 25: Estimates of female grizzly bears for the BMA 6 DNA mark-recapture grid area and Waterton National Park. Densities (bears/1000 km^2^) are based on total area of the DNA grid as listed in Table 1.

| Model |  | Expected Population size | | | |  | Density | |  |  |
| --- | --- | --- | --- | --- | --- | --- | --- | --- | --- | --- |
| Density | Detection | $\hat{N}$ | SE | Conf. Int. | | CV | $\hat{D}$ | SE | Conf. Int. | |
| RISK | g_0_(TRI) σ (.) | 14.3 | 3.7 | 8.8 | 23.5 | 25.6% | 5.06 | 1.31 | 3.11 | 8.31 |
| Constant | g_0_(TRI) σ (.) | 24.0 | 7.3 | 13.5 | 42.9 | 30.2% | 8.49 | 2.58 | 4.77 | 15.17 |
| RISK | constant | 14.6 | 4.1 | 8.4 | 25.1 | 28.4% | 5.16 | 1.45 | 2.97 | 8.88 |
| Closed N/Telemetry | | 32.7 | 10.3 | 19.4 | 62.6 | 31.6% | 11.55 | 11.57 | 3.64 | 6.86 |

### Males

As with the female bears, males were mainly detected on the very western edge of the sampling grid with one movement event to the east (Figure 19). Overall, 19 bears were identified with only 25 detection events over the 4 sampling sessions. The mean distance moved between detections was 8.9 km with and RSPV of 8.9 km for the 25 detection events.


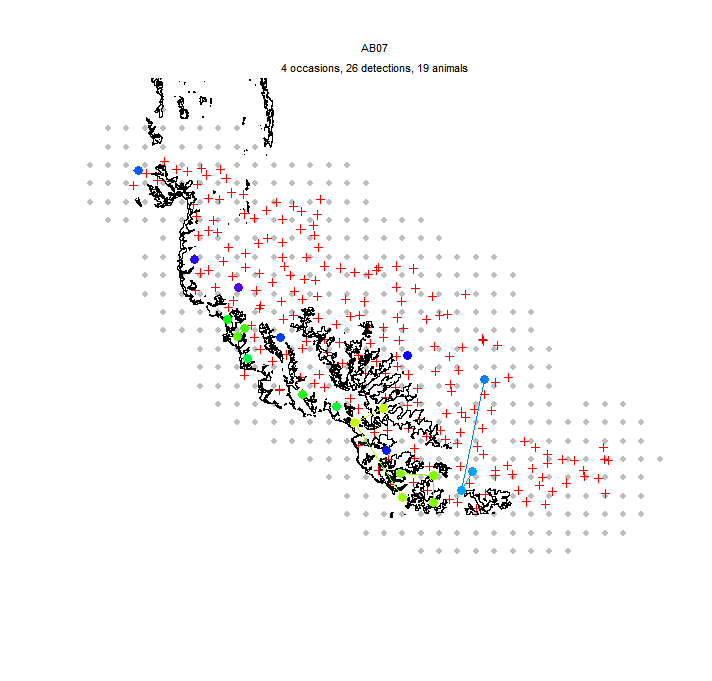


Figure 19: : Detections and tracks of male grizzly bears in BMA 6 from repeated detections relative to habitat mask (grey dots), hair snag site locations (red + signs) and barren non-habitat (black polygons with no mask points inside them). The actual sequence of tracks from repeated detections does not necessarily reflect the true path of movements given that the time of within-session detection at hair snag sites is unknown. The cumulative locations of HS sites are displayed which include sites moved each session. Therefore, the actual spatial coverage of HS sites for one session will be overrepresented by this graphic. Map was produced using the *secr* package ^6^ (v 2.9.2; <http://www.otago.ac.nz/density/SECRinR.html>) in program R^10^ (v 3.1.2; [www.r-project.org](http://www.r-project.org) ) plotting functions.

Model selection results were similar to female grizzly bears with detection probabilities at the home range center most influenced by terrain ruggedness (Table 26, model 2). Of the density surface models, a model with risk as a predictor of density was most supported (Table 26, model 1).

Table 26 Abridged model selection for male grizzly bears for the BMA 6 (2007) inventory. AIC_c_ = sample size adjusted Akaike Information Criterion, ΔAIC_c_ = the difference in AIC_c_ between the model and the most supported model, AIC_c_ weight = w_i_, K, the number of model parameters and log-likelihood are given. Baseline constant models are shaded for reference with covariate models. The most supported baseline model (of models considered as listed in Table 2) is shown.

| No | Density | Detection | AICc | ΔAIC_c_ | w_i_ | K | LL |
| --- | --- | --- | --- | --- | --- | --- | --- |
| 1 | Risk | g_0_(TRI) σ (.) | 203.8 | 0.00 | 0.48 | 5 | -94.6 |
| 2 | constant | g_0_(TRI) σ (.) | 204.2 | 0.48 | 0.37 | 4 | -96.7 |
| 3 | RSF | g_0_(TRI) σ (.) | 207.4 | 3.63 | 0.08 | 5 | -96.4 |
| 4 | RSF +Risk | g_0_(TRI) σ (.) | 207.9 | 4.17 | 0.06 | 6 | -94.5 |
| 5 | RSF +Risk +RSF*Risk | g_0_(TRI) σ (.) | 212.1 | 8.36 | 0.01 | 7 | -94.0 |
| 6 | constant | constant | 219.5 | 15.79 | 0.00 | 3 | -106.0 |

A model with Risk as a density covariate but with constant detection parameters (g_0_(.) σ(.)) also showed support from the data set (AICc=203.2) suggesting only weak support for the TRI covariate for males once Risk was included as a density covariate. We suspect this result was partially due to the sparse data for males in BMA 6 (Table 23) and subsequent limitations on model complexity.

A plot of predicted density as a function of risk suggested densities were mainly at areas of risk less than 0.3 (Figure 20).

Figure 20: Estimates of density as a function of risk from Model 1 (Table 13)

Estimates were also run at 2 km mask spacing to check if the relatively large 3.5 km spacing affected estimates. Estimates from the D(.) g_0_(TRI) σ (.) for the DNA sampling grid was 35.8 (SE=13.6, CI=17.5-73.5) compared to the estimate of 37.5 for the 3.5 km mask (Table 27). These estimates were slightly different than the 3.5 km grid spacing suggesting that mask spacing has a marginal effect on estimates

Table 27: Estimates of male grizzly bears for the BMA 6 2007 DNA mark-recapture grid area and Waterton National Park. Densities (bears/1000 km^2^) are based on total area of the DNA grid as listed in Table 1. The first model and estimates listed for each area is from the most supported SECR model. Other models and estimates are given to explore sensitivity to model assumptions.

| SECR model | | Expected population size | | |  |  | Density | |  |  |
| --- | --- | --- | --- | --- | --- | --- | --- | --- | --- | --- |
| Density | Detection | $\hat{N}$ | SE | Conf. Int. | | CV | $\hat{D}$ | SE | Conf. Int. | |
| RISK | g_0_(TRI) σ (.) | 21.3 | 7.0 | 11.3 | 40.0 | 37.3% | 7.53 | 2.49 | 4.01 | 14.15 |
| Constant | g_0_(TRI) σ (.) | 37.5 | 14.3 | 18.2 | 77.4 | 38.2% | 13.26 | 5.06 | 6.44 | 27.37 |
| RISK | constant | 20.0 | 6.2 | 11.0 | 36.3 | 36.7% | 7.07 | 2.20 | 3.89 | 12.84 |
| Closed N/Telemetry | | 17.7 | 17.7 | 4.9 | 12.6 | 34.3 | 27.5% | 6.26 | 1.73 | 4.46 |

# Summary of model parameters

Tables 28 and 29 provide estimates of the beta parameters for each of the density surface models used in the analysis.

Table 28: Beta parameters for most supported density surface models for females. Wald tests (β/SE(β)) for individual parameters are also given. Density (D) and scale (σ) are on the log link scale whereas g_0_ is on the logit scale.

| BMA | Parameter | Covariate | β | SE | Conf. Limit | | Wald | Z-score |
| --- | --- | --- | --- | --- | --- | --- | --- | --- |
| 2 | D | Intercept | -11.53 | 0.54 | -12.58 | -10.48 | -21.54 | 0.000 |
|  |  | RSF | 19.47 | 3.28 | 13.05 | 25.89 | 5.94 | 0.000 |
|  |  | RISK | 3.48 | 1.47 | 0.61 | 6.35 | 2.37 | 0.018 |
|  |  | RSF*RISK | -27.20 | 11.49 | -49.72 | -4.68 | -2.37 | 0.018 |
|  | g_0_ | Intercept | -1.19 | 0.19 | -1.56 | -0.81 | -6.17 | 0.000 |
|  |  | CC | -0.16 | 0.05 | -0.27 | -0.06 | -3.08 | 0.002 |
|  | σ | Intercept | 8.52 | 0.06 | 8.40 | 8.63 | 142.02 | 0.000 |
|  |  | TRI | -0.08 | 0.03 | -0.14 | -0.02 | -2.45 | 0.014 |
| 3 | D | Intercept | -14.39 | 0.94 | -16.23 | -12.55 | -15.35 | 0.000 |
|  |  | RSF | 25.46 | 5.12 | 15.42 | 35.50 | 4.97 | 0.000 |
|  | g_0_ | Intercept | -2.82 | 0.27 | -3.34 | -2.30 | -10.60 | 0.000 |
|  |  | TRI | 0.76 | 0.23 | 0.32 | 1.21 | 3.36 | 0.001 |
|  | σ | Intercept | 8.84 | 0.08 | 8.68 | 9.00 | 108.40 | 0.000 |
| 4 | D | Intercept | -9.69 | 0.12 | -9.92 | -9.45 | -80.01 | 0.000 |
|  |  | RSF | 1.32 | 0.36 | 0.62 | 2.02 | 3.70 | 0.000 |
|  | g_0_ | Intercept | -2.46 | 0.56 | -3.56 | -1.36 | -4.37 | 0.000 |
|  |  | TRI | 0.42 | 0.23 | -0.03 | 0.86 | 1.83 | 0.067 |
|  | σ | Intercept | 8.46 | 0.12 | 8.21 | 8.70 | 68.25 | 0.000 |
|  |  | T | 0.12 | 0.05 | 0.03 | 0.21 | 2.54 | 0.011 |
| 5 | D | Intercept | -8.64 | 0.36 | -9.35 | -7.93 | -23.80 | 0.000 |
|  |  | RISK | -4.16 | 1.64 | -7.37 | -0.94 | -2.53 | 0.011 |
|  | g_0_ | Intercept | -1.91 | 0.29 | -2.48 | -1.35 | -6.64 | 0.000 |
|  |  | T | 0.18 | 0.11 | -0.04 | 0.39 | 1.63 | 0.104 |
|  | σ | Intercept | 8.50 | 0.08 | 8.35 | 8.66 | 105.85 | 0.000 |
| 6 | D | Intercept | -6.94 | 0.64 | -8.20 | -5.69 | -10.84 | 0.000 |
|  |  | RISK | -7.62 | 2.50 | -12.51 | -2.72 | -3.05 | 0.002 |
|  | g_0_ | Intercept | -1.38 | 1.39 | -4.10 | 1.34 | -0.99 | 0.320 |
|  |  | TRI | 0.65 | 0.57 | -0.46 | 1.76 | 1.15 | 0.251 |
|  | σ | Intercept | 7.77 | 0.16 | 7.46 | 8.07 | 49.66 | 0.000 |

Table 29: Beta parameters for the most supported density surface models for males. Wald tests (β/SE(β)) for individual parameter significance are also given. Density (D) and scale (σ) are on the log link scale whereas g_0_ is on the logit scale.

| BMA | Parameter | Covariate | β | SE | Conf. Limit. | | Wald | Z-score |
| --- | --- | --- | --- | --- | --- | --- | --- | --- |
| 2 | D | Intercept | -12.21 | 0.83 | -13.84 | -10.59 | -14.72 | 0.000 |
|  |  | RSF | 21.73 | 5.22 | 11.50 | 31.96 | 4.16 | 0.000 |
|  |  | RISK | 3.87 | 2.29 | -0.63 | 8.36 | 1.69 | 0.092 |
|  |  | RSF*RISK | -37.13 | 18.74 | -73.86 | -0.39 | -1.98 | 0.048 |
|  | g_0_ | Intercept | -3.22 | 0.18 | -3.58 | -2.86 | -17.64 | 0.000 |
|  |  | TRI | 0.53 | 0.18 | 0.18 | 0.88 | 3.00 | 0.003 |
|  | σ | Intercept | 9.13 | 0.05 | 9.03 | 9.22 | 188.33 | 0.000 |
| 3 | D | Intercept | -14.90 | 0.91 | -16.69 | -13.11 | -16.32 | 0.000 |
|  |  | RSF | 24.19 | 4.19 | 15.98 | 32.39 | 5.78 | 0.000 |
|  | g_0_ | Intercept | -2.37 | 0.21 | -2.78 | -1.96 | -11.25 | 0.000 |
|  | σ | Intercept | 9.33 | 0.13 | 9.09 | 9.58 | 74.35 | 0.000 |
|  |  | T | -0.27 | 0.06 | -0.39 | -0.16 | -4.52 | 0.000 |
|  |  | TRI | 0.18 | 0.08 | 0.02 | 0.34 | 2.17 | 0.030 |
| 4 | D | Intercept | -14.74 | 1.77 | -18.21 | -11.26 | -8.30 | 0.000 |
|  |  | RSF | 19.15 | 8.31 | 2.87 | 35.43 | 2.31 | 0.021 |
|  | g_0_ | Intercept | -3.26 | 0.34 | -3.93 | -2.59 | -9.58 | 0.000 |
|  |  | TRI | 0.31 | 0.16 | -0.01 | 0.63 | 1.92 | 0.055 |
|  | σ | Intercept | 9.46 | 0.08 | 9.29 | 9.62 | 112.72 | 0.000 |
| 5 | D | Intercept | -8.74 | 0.38 | -9.48 | -7.99 | -23.01 | 0.000 |
|  |  | Risk | -5.24 | 1.78 | -8.74 | -1.75 | -2.94 | 0.003 |
|  | g_0_ | Intercept | -2.87 | 0.24 | -3.34 | -2.40 | -12.04 | 0.000 |
|  | σ | Intercept | 10.65 | 0.25 | 10.16 | 11.14 | 42.76 | 0.000 |
|  |  | H1 | -1.49 | 0.26 | -2.00 | -0.99 | -5.81 | 0.000 |
|  |  | Mixture | 4.60 | 1.03 | 2.59 | 6.62 | 4.47 | 0.000 |
| 6 | D | Intercept | -5.97 | 0.77 | -7.47 | -4.46 | -7.79 | 0.000 |
|  |  | RISK | -14.48 | 5.80 | -25.86 | -3.11 | -2.50 | 0.013 |
|  | g_0_ | Intercept | -3.59 | 0.90 | -5.37 | -1.82 | -3.97 | 0.000 |
|  |  | TRI | 0.30 | 0.28 | -0.24 | 0.85 | 1.09 | 0.277 |
|  | σ | Intercept | 8.91 | 0.20 | 8.52 | 9.30 | 44.71 | 0.000 |

# Literature cited

1 AlbertaGrizzlyBearInventoryTeam. Grizzly bear population size and density estimates for the 2008 DNA Inventory of the Grande Cache Bear Management Area (BMA 2). (Alberta Sustainable Resource Development, Fish and Wildlife Division, 2009).

2 Boulanger, J. *et al.* 2004 Population inventory and density estimates for the Alberta 3B and 4B Grizzly Bear Management Area. (Alberta Sustainable Resource Development, Hinton, Alberta, 2005).

3 Boulanger, J. *et al.* Grizzly bear population and density estimates for the 2005 Alberta Unit 4 Management Area Inventory. (Alberta Sustainable Resource Development, Fish and Wildlife Division, Hinton, AB, 2005).

4 AlbertaGrizzlyBearInventoryTeam. Grizzly bear population and density estimates for the 2006 Alberta Unit 5 Management Area inventory., (Alberta Sustainable Resource Development, Fish and Wildlife Division, Hinton, Alberta, 2007).

5 GrizzlyBearInventoryTeam. Grizzly Bear Population and Density Estimates for Alberta Bear Management Unit 6 and British Columbia Management Units 4-1, 4-2, and 4-23 (2007) (Alberta Sustainable Resource Development, Fish and Wildlife Division, British Columbia Ministry of Forests and Range, British Columbia Ministry of Environment, and Parks Canada, Hinton Alberta 2008).

6 Efford, M. G. secr spatially explicit capture recapture models. R package version 2.8.1, <http://CRAN.R-project.org/package=secr>. (2014).

7 Riley, S. J., DeGloria, S. D. & Elliot, R. A terrain ruggedness index that quantifies topographic heterogeneity,. *Intermountain Journal of Sciences* **5**, 1-4 (1999).

8 Pledger, S. & Efford, M. Correction of bias due to heterogeneous capture probabilities in capture-recapture studies of open populations. *Biometrics* **54**, 888-898 (1998).

9 Swikhart, R. K. & Slade, N. A. On testing for the independence of animal movements. *J. of Agricultural, Biological, and Environmental Statistics* **2**, 48-63 (1997).

10 R: A language and environment for statistical computing (R Foundation for Statistical Computing, Vienna, Austria, 2009).

11 Boulanger, J. *et al.* An empirical test of DNA mark-recapture sampling strategies for grizzly bears. *Ursus* **17**, 149-158 (2006).

# Appendix S2: Information on estimation of density using closed models and radio telemetry

**An assessment of spatial mark-recapture as a conservation tool for monitoring grizzly bear populations in Alberta**

**John Boulanger^1,*^, Scott E. Nielsen^2+^, and Gord Stenhouse^3+^**

^1^ Integrated Ecological Research, 924 Innes St., Nelson, BC V1L 5T2 Canada

^2^ Department of Renewable Resources, University of Alberta, 751 General Services Building, Edmonton, AB T6G 2H1, Canada

^3^ fRI Research 1176 Switzer Drive, Hinton, Alberta, T7V 1V3, Canada

*** boulange@ecological.bc.ca

We used the closed N/Telemetry estimator^12-14^ in program MARK^15^ to estimate density and average number of bears on each of the BMA sampling grids. The Closed N/Telemetry density estimation model of Ivan (2013a) in program MARK (White and Burnham 1999) produces density estimates using a modified Huggins (1991) estimator of population size where counts of detected individuals are replaced by estimates of residency derived from radio collared bears (symbolized as $\tilde{p}$) as estimated by the proportion of telemetry points that a radio collared bear spent on the sample grid during the time period that sampling occurred. The average number of bears on the sample grid is estimated as (N_ave_) ($\hat{N}_{ave}= \sum_{i=1}^{M_{t+1}} (\frac{\tilde{p}_{i}}{p_{i}^{*}})$) where
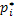
$p_{i}^{*}$, is the probability of detection of each individual (*i*) across sessions.
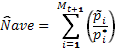
 Density is estimated by dividing N_ave_ by the area of the sample grid. Residency of radioed or DNA bears often is a function of the distance of the bears from the sampling grid edge^16^. Bears that occur near the edge are more likely to be off the grid than bears in the middle. We therefore estimated the distance of radioed and DNA bears from the entire grid edge. Only radioed collared bear points that fell within the DNA sampling grid were used to estimate mean locations and distance from edge to ensure equivalency of DNA and radio collared bears. By using distance from edge as a covariate, the assumption of similar distributions of DNA and radio collared bears was relaxed, making this assumption more reasonable. Data from radio collared bears was available for years previous to the actual DNA project. This data was used for the analysis by adding the radio collared bears as a unique group in MARK. This group was used to only estimate $\tilde{p}$ under the assumption of similar residency of grizzly bears prior to the DNA project and during the project. Only one record was used for each collared bear in the analysis to prevent issues with pseudoreplication. The yearly record that occurred closest to the year of the DNA project was used when more than one year of telemetry data was used for a bear.

The number of collared bears varied by BMA with low sample sizes, especially for males, in some BMA’s (Table 28). Logistic regression, as well as Ivan model analyses, were used to test pooling strategies for BMA’s when sample sizes were low. The historic inventory analyses occurred before the Ivan estimator and used the proportion of radio collared points on the grid for each bear^17^ or the core method^16^ to estimate bear density on the sampling grid.

Table 30: Summary of bears detected using DNA mark-recapture, collared bears used to estimate residency, and citations for further information on each project. The citations are available online from the government of Alberta (<http://aep.alberta.ca/fish-wildlife/wildlife-management/grizzly-bear-recovery-plan/>) using the links in the literature cited section.

|  |  |  | DNA bears | | Collared bears | |  |
| --- | --- | --- | --- | --- | --- | --- | --- |
| BMA | Area of DNA grid | Year | F | M | F | M | Source of information |
| 2 | 19,502 | 2008 | 161 | 108 | 23 | 37 | Inventory report^1^ |
| 3 | 8,820 | 2004 | 24 | 20 | 38 | 33 | Inventory report^2^ |
| 4 | 9,016 | 2005 | 25 | 17 | 11 | 2 | Inventory report^3^ |
| 5 | 8,134 | 2006 | 35 | 33 | 19 | 18 | Inventory report^4^ |
| 6 | 2,827 | 2007 | 13 | 19 | 6 | 4 | Inventory report^5^ |

For closed model formulation, we used results from individual analyses of yearly BMA inventory projects to parameterize closed models for the analyses as summarized in Table 29.

Table 31: Summary of supported covariates for closed model selection from individual analyses of bear management areas. Citations that provide further details on each analysis are given in Table 1.

| BMA (*A*) | Year | covariate | explanation |
| --- | --- | --- | --- |
| 2 | 2008 | sex | sex of bear |
|  |  | fix | whether sites were fixed or moved |
|  |  | occ | whether cell size was 7x7 or 14X14 km |
|  |  | pgen | genotyping success per session |
| 3 | 2004 | t4 | different detection for session 4 |
|  |  | ld | detection as a function of log distance from grid edge (d) |
|  |  | pi | undefined heterogeneity (using mixture models) |
| 4 | 2005 | t1 | different detection for session 1 |
|  |  | ld | detection as a function of log distance from grid edge (d) |
| 5 | 2006 | TF | temporal trend for females |
|  |  | sex | sex of bear |
| 6 | 2007 | sex | sex of bear |
|  |  | pi | undefined heterogeneity |

Model selection for the closed model/telemetry analysis initially focused on parsimonious models for predicting residence of bears given low sample sizes for some of the bear management areas (Table 30). For example, only 2 and 4 yearly locations were available for males in BMA’s 4 and 6 (Table 28) which made modelling of residency as a function of distance from grid edge problematic for these areas. In BMA 6, residency for males was variable as a function of distance from edge, however, low sample sizes of points (4) precluded solid evaluation of trend in distance from edge. Therefore, models with constant residency (-M6) for this BMA (model 7) were contrasted with models that pooled data from other BMA’s to model residency as a function of distance from edge (Models 8-11). For example, model 10 pooled data for males from BMA’s 4, 5, and 6 to model residency as a function of distance from grid edge. Of models considered, a model that had BMA and sex-specific relationships for residency as a function of distance from edge with residency held constant for males in BMA 6 was most supported (Model 7). Models were then considered that used mixture models to model undefined heterogeneity (Model 5) with covariate models from individual analyses for each BMA (Table 2: Models 1-4). Detection probability covariate models (as defined in Table 2) were more supported than mixture models. Detection probability as a function of distance from edge was then considered. Mixture models with distance from edge terms for DNA bears did not converge. A model with sex and BMA-specific relationships (Model 4) was compared with sex-specific (Model 3) and pooled relationships (models 1 and 2) between detection probability and distance from grid edge. Of these, a model with sex-specific relationships for the pooled data from BMA’s 3, 4, 5, and 6 was most supported with no relationship between distance from edge and detection for BMA 2 was most supported (Model 1).

Table 32: Closed model/telemetry model selection for Alberta BMA grizzly bear inventory data 2004-8. Detection probability is dichotomized by base variation and variation as a function of distance from edge. Residency pertains to the analysis of radio collar data. The symbol A pertains to BMA-specific variation with the BMA number listed after A when variation occurred within a subset of the BMA’s. A legend for covariate terms is given in Table 2. AIC_c_ = sample size adjusted Akaike Information Criterion, ΔAIC_c_ = the difference in AIC_c_ between the model and the most supported model, AIC_c_ weight = w_i_, K, the number of model parameters and log-likelihood (*LL*) are given.

| No | Detection probability (*p*) | *p*(Dist. from edge) | Residency ($\tilde{p}$) | AICc | ∆AICc | w_i_ | K | Deviance |
| --- | --- | --- | --- | --- | --- | --- | --- | --- |
| 1 | A+sex+A2(fix*sex+res+geno)+A3(t4)+A4(t1)+A5(TF) | sex*ld(A3456) | sex*A*ld (-M6) | 12494.0 | 0.0 | 0.72 | 33 | 12427.1 |
| 2 | A+sex+A2(fix*sex+res+geno)+A3(t4)+A4(t1)+A5(TF) | sex*ld (A3456,A2) | sex*A*ld (-M6) | 12497.1 | 3.1 | 0.16 | 35 | 12426.1 |
| 3 | A+sex+A2(fix*sex+res+geno)+A3(t4)+A4(t1)+A5(TF) | sex*ld | sex*A*ld (-M6) | 12497.5 | 3.6 | 0.12 | 33 | 12430.7 |
| 4 | A+sex+A2(fix*sex+res+geno)+A3(t4)+A4(t1)+A5(TF) | sex*A*ld | sex*A*ld (-M6) | 12507.0 | 13.0 | 0.00 | 41 | 12423.6 |
| 5 | pi(A) p1&2(A) |  | sex*A*ld (-M6) | 12514.0 | 20.0 | 0.00 | 31 | 12451.2 |
| 6 | A+sex | sex*ld | sex*A*ld (-M6) | 12517.0 | 23.0 | 0.00 | 26 | 12464.5 |
| 7 | A+sex |  | sex*A*ld (-M6) | 12521.2 | 27.2 | 0.00 | 24 | 12472.7 |
| 8 | A+sex A | sex*ld | sex*A*ld (A6) | 12588.8 | 94.8 | 0.00 | 27 | 12534.2 |
| 9 | A+sex | sex*ld | sex*A*ld (A56*sex) | 12598.1 | 104.2 | 0.00 | 26 | 12545.6 |
| 10 | A+sex | sex*ld | sex*A*ld(A456*M) | 12605.6 | 111.6 | 0.00 | 26 | 12553.0 |
| 11 | A+sex | sex*ld | sex*A*ld(A3456*sex) | 12633.8 | 139.8 | 0.00 | 22 | 12589.4 |
| 12 | A+sex | sex*ld | sex*A*d | 12834.8 | 340.8 | 0.00 | 28 | 12778.2 |
| 13 | A+sex | sex*ld | sex+A+sex*ld | 13099.2 | 605.2 | 0.00 | 16 | 13067.0 |
| 14 | pi(A*sex) p1&2(A*sex) |  | A*sex | 14309.1 | 1815.1 | 0.00 | 33 | 14242.2 |
| 15 | pi(A) p1&2(A) |  | A*sex | 14994.0 | 2500.1 | 0.00 | 23 | 14947.6 |
| 16 | p(A*year*t) |  | A*sex | 15004.8 | 2510.8 | 0.00 | 50 | 14902.8 |
| 17 | pi(sex) pi(A*sex) |  | A*sex | 15007.3 | 2513.3 | 0.00 | 31 | 14945.5 |
| 18 | A+sex | sex*ld | constant | 15657.6 | 3163.6 | 0.00 | 9 | 15639.5 |

The relationship between residency and distance from edge for Model 1 (Table 3) showed variable relationships for males and females and BMA’s (Figure 21). The coverage of radio collared bears (blue points indicating residence for each collared bear) was relatively similar to DNA bears (the circles on each prediction line).


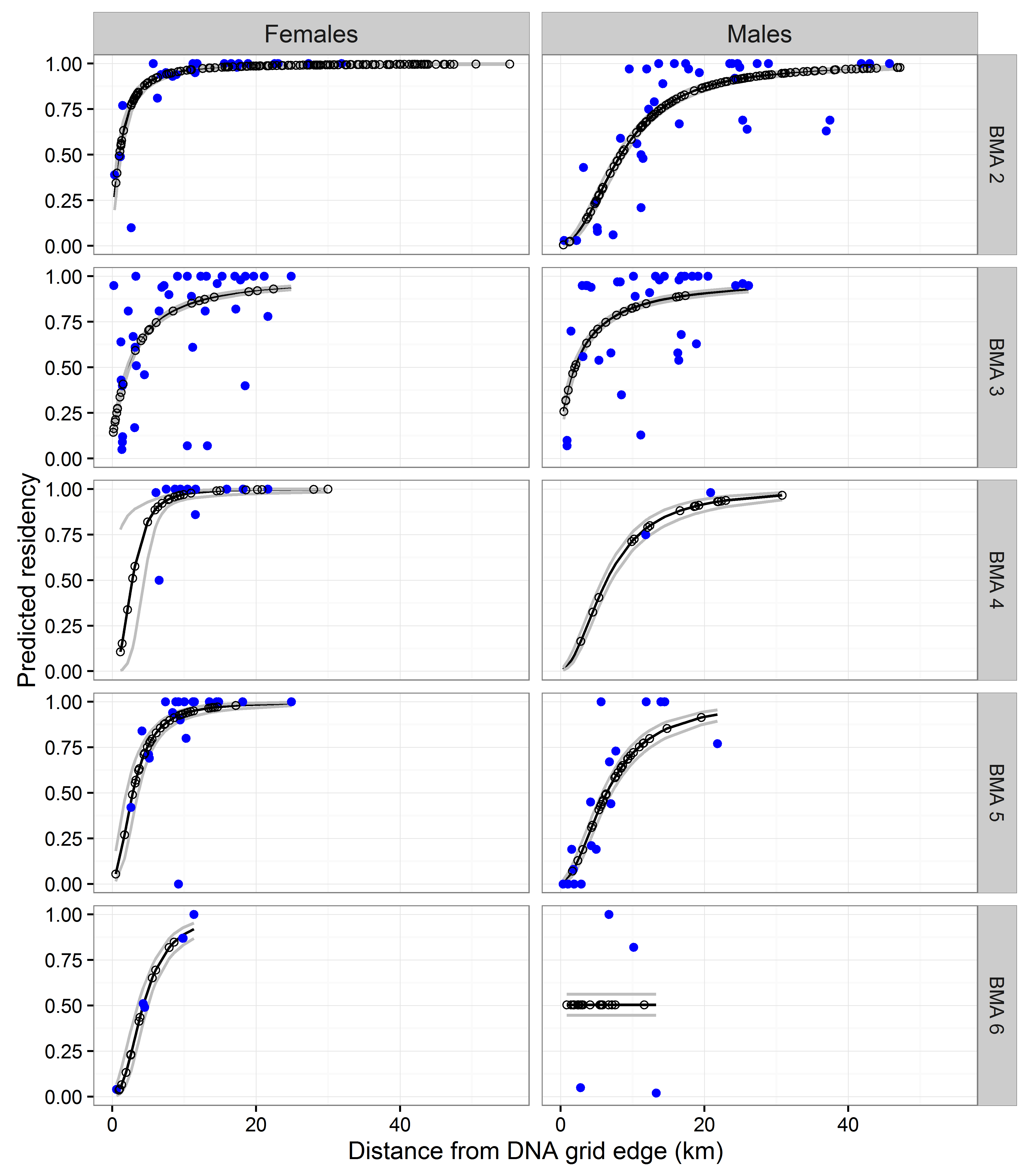


Figure 21: The predicted relationship between residency and distance from grid edge from analysis of radio collared bear data from Model 1 (Table 3). Blue dots are the observed residency for each collared bear (number of locations on grid/number of locations total). The circles on each prediction line indicate mean distances from edge for bears detected using DNA methods. The closed N/telemetry model assigned residency for each DNA bear based on the prediction lines for each BMA.

Model averaged estimates of density and average number of bears on the sampling grid is given in Table 31.

Table 33: Model averaged estimates of density and average number of bear on the sampling grid from closed model/Telemetry analysis of Alberta Bear Management Area inventories (2004-8).

| BMA | sex | Density | SE | Conf. Interval | | CV | Ave N | SE | Conf. Interval | |
| --- | --- | --- | --- | --- | --- | --- | --- | --- | --- | --- |
| 2 | F | 10.07 | 0.63 | 8.91 | 11.39 | 6.3% | 196.6 | 12.3 | 173.9 | 222.3 |
|  | M | 5.17 | 0.49 | 4.29 | 6.23 | 9.5% | 101.0 | 9.6 | 83.8 | 121.7 |
|  | P | 15.24 | 0.81 | 13.73 | 16.92 | 5.3% | 297.6 | 15.8 | 268.2 | 330.3 |
| 3 | F | 1.73 | 0.21 | 1.36 | 2.20 | 12.3% | 14.4 | 1.8 | 11.3 | 18.3 |
|  | M | 1.68 | 0.20 | 1.34 | 2.11 | 11.6% | 14.0 | 1.6 | 11.1 | 17.5 |
|  | P | 3.42 | 0.29 | 2.89 | 4.03 | 8.5% | 28.4 | 2.4 | 24.0 | 33.5 |
| 4 | F | 2.37 | 0.19 | 2.02 | 2.78 | 8.2% | 21.3 | 1.7 | 18.1 | 25.0 |
|  | M | 1.55 | 0.10 | 1.35 | 1.76 | 6.8% | 13.9 | 0.9 | 12.2 | 15.9 |
|  | P | 3.91 | 0.22 | 3.51 | 4.37 | 5.6% | 35.2 | 2.0 | 31.5 | 39.3 |
| 5 | F | 4.25 | 0.51 | 3.36 | 5.38 | 12.0% | 34.6 | 4.2 | 27.4 | 43.8 |
|  | M | 3.10 | 0.67 | 2.04 | 4.71 | 21.6% | 25.2 | 5.4 | 16.6 | 38.3 |
|  | P | 7.35 | 0.84 | 5.88 | 9.20 | 11.5% | 59.8 | 6.9 | 47.8 | 74.8 |
| 6 | F | 4.20 | 2.65 | 1.35 | 13.06 | 63.1% | 11.9 | 7.5 | 3.8 | 36.9 |
|  | M | 8.06 | 3.66 | 3.45 | 18.84 | 45.4% | 22.8 | 10.4 | 9.8 | 53.3 |
|  | P | 12.26 | 4.52 | 6.09 | 24.69 | 36.9% | 34.7 | 12.8 | 17.2 | 69.8 |

**Literature cited**

1 AlbertaGrizzlyBearInventoryTeam. Grizzly bear population size and density estimates for the 2008 DNA Inventory of the Grande Cache Bear Management Area (BMA 2). (Alberta Sustainable Resource Development, Fish and Wildlife Division, 2009).

2 Boulanger, J. *et al.* 2004 Population inventory and density estimates for the Alberta 3B and 4B Grizzly Bear Management Area. (Alberta Sustainable Resource Development, Hinton, Alberta, 2005).

3 Boulanger, J. *et al.* Grizzly bear population and density estimates for the 2005 Alberta Unit 4 Management Area Inventory. (Alberta Sustainable Resource Development, Fish and Wildlife Division, Hinton, AB, 2005).

4 AlbertaGrizzlyBearInventoryTeam. Grizzly bear population and density estimates for the 2006 Alberta Unit 5 Management Area inventory., (Alberta Sustainable Resource Development, Fish and Wildlife Division, Hinton, Alberta, 2007).

5 GrizzlyBearInventoryTeam. Grizzly Bear Population and Density Estimates for Alberta Bear Management Unit 6 and British Columbia Management Units 4-1, 4-2, and 4-23 (2007) (Alberta Sustainable Resource Development, Fish and Wildlife Division, British Columbia Ministry of Forests and Range, British Columbia Ministry of Environment, and Parks Canada, Hinton Alberta 2008).

6 Efford, M. G. secr spatially explicit capture recapture models. R package version 2.8.1, <http://CRAN.R-project.org/package=secr>. (2014).

7 Riley, S. J., DeGloria, S. D. & Elliot, R. A terrain ruggedness index that quantifies topographic heterogeneity,. *Intermountain Journal of Sciences* **5**, 1-4 (1999).

8 Pledger, S. & Efford, M. Correction of bias due to heterogeneous capture probabilities in capture-recapture studies of open populations. *Biometrics* **54**, 888-898 (1998).

9 Swikhart, R. K. & Slade, N. A. On testing for the independence of animal movements. *J. of Agricultural, Biological, and Environmental Statistics* **2**, 48-63 (1997).

10 R: A language and environment for statistical computing (R Foundation for Statistical Computing, Vienna, Austria, 2009).

11 Boulanger, J. *et al.* An empirical test of DNA mark-recapture sampling strategies for grizzly bears. *Ursus* **17**, 149-158 (2006).

12 Ivan, J. S. *Density, demography, and seasonal movements of snowshoe hares in central Colorado* Ph.D. thesis, Ph.D Dissertation, Colorado State University, (2011).

13 Ivan, J. S., White, G. C. & Shenk, T. M. Using auxiliary telemetry information to estimate animal density from capture-recapture data. *Ecology* **94**, 809-816 (2013).

14 Ivan, J. S., White, G. C. & Shenk, T. M. Using simulation to compare methods for estimating density from capture-recapture data. *Ecology* **94**, 817-826 (2013).

15 White, G. C. & Burnham, K. P. Program MARK: Survival estimation from populations of marked animals. *Bird Study Supplement* **46**, 120-138 (1999).

16 Boulanger, J. & McLellan, B. Closure violation in DNA-based mark-recapture estimation of grizzly bear populations. *Canadian Journal of Zoology* **79**, 642-651 (2001).

17 White, G. C. & Shenk, T. M. in *Design and Analysis of Radio Telemetry Studies* (eds J.J. Millspaugh & J.M. Marzluff) (Academic Press, 2001).
